# Full-length HLA sequencing in adult T cell leukemia–lymphoma uncovers multiple gene alterations

**DOI:** 10.1038/s41375-021-01403-1

**Published:** 2021-09-13

**Authors:** Keita Tamaki, Satoko Morishima, Shingo Suzuki, Atsuko Shigenari, Ikumi Nomura, Yutaro Yokota, Kazuho Morichika, Yukiko Nishi, Sawako Nakachi, Shiki Okamoto, Takuya Fukushima, Takashi Shiina, Hiroaki Masuzaki

**Affiliations:** 1 Division of Endocrinology, Diabetes and Metabolism, Hematology and Rheumatology, Second Department of Internal Medicine, Graduate School of Medicine, University of the Ryukyus, Nishihara, Okinawa Japan; 2 Division of Basic Medical Science and Molecular Medicine, Department of Molecular Life Science, Tokai University School of Medicine, Isehara, Kanagawa Japan; 3 Department of Medicine, Faculty of Medicine, University of the Ryukyus, Nishihara, Okinawa Japan; 4 Laboratory of Hematoimmunology, School of Health Sciences, Faculty of Medicine, University of the Ryukyus, Nishihara, Okinawa Japan

**Keywords:** T-cell lymphoma, Cancer genomics

**To the Editor:**

Adult T cell leukemia–lymphoma (ATL) is a peripheral T cell lymphoid malignancy caused by human T cell leukemia virus type 1 (HTLV-1). The clinical subtypes of ATL are closely associated with its prognosis, which is extremely poor in aggressive subtypes (acute, lymphoma, and unfavorable chronic) compared to indolent subtypes (favorable chronic and smoldering) [1].

Human leukocyte antigen (HLA) plays an important role in T cell-mediated elimination of cancer cells. Downregulation of HLA occurs in various cancers and has been linked to poor prognosis [2]. Structural defects in the HLA molecule are usually associated with loss of heterozygosity (LOH) and somatic mutations in HLA genes [3]. However, the detection of LOH and somatic mutations in cancer cells seems to be extremely difficult due to the highly polymorphic nature of HLA genes.

Conventional polymerase chain reaction (PCR)-based HLA typing mainly focuses on the polymorphic exons encoding the antigen recognition domains. Therefore, the genetic variations in the non-coding regions or in the exons outside of the polymorphic exons have largely remained ignored. In addition, methods for precisely deciphering HLA alleles are limited due to chromosomal phase ambiguity. To overcome a line of difficulties, we successfully developed the super high-resolution single-molecule sequence-based typing (SS-SBT) method [4], which combines long-range PCR amplification and next-generation sequencing (NGS). This method provides *in phase* high-resolution typing, which includes nucleotide differences in both the coding and non-coding regions of HLA genes [5]. Here, we used the SS-SBT method to investigate the entire region of HLA genes in both ATL and non-ATL cells obtained from the same patients.

We evaluated 25 patients diagnosed as ATL between 2012 and 2018. Their characteristics are summarized in Supplementary Tables 1 and 2. Of all the patients, five had chronic-type ATL; the remaining 20 had acute-type ATL. Because cell adhesion molecule 1 (CADM1) is known to be expressed ectopically in ATL cells [6], we separated peripheral blood mononuclear cells (PBMCs) obtained from patients into CADM1-positive ATL cells and CADM1-negative non-ATL cells. We analyzed eight classical HLA loci in ATL and non-ATL cells from the same patients. Further materials and methods are shown in the Supplemental Methods.

Through magnetic cell sorting, more than 97% pure CADM1-positive cells (ATL cells) and more than 90% CADM1-negative cells (non-ATL cells) were isolated in CADM1-positive and -negative fractions, respectively (Supplementary Table 2). After the sequencing of eight HLA loci using the genomic DNA from ATL and non-ATL cells, basic sequence read information was obtained (Supplementary Table 3). The HLA typing results of all of the patients are shown in Supplementary Table 4. A total of 11 novel HLA alleles were identified in non-ATL cells. All of the novel variants were located in intronic regions or in the 3′UTR (Supplementary Table 5).

To detect somatic mutations in HLA genes, HLA allele sequences were determined in ATL cells. Mutational events were observed in the ATL cells but not in the non-ATL cells. We found a total of 18 somatic mutations in ATL cells from 8 patients, including 13 single nucleotide variants (SNVs) and five insertions/deletions (indels) (Table 1). Of the 13 SNVs, three were nonsense mutations, seven were nonsynonymous mutations, and two were mutations at splice sites. There was only one mutation located in intron 1 of HLA-A. Mutations in the splice sites in intron 2 caused frameshift-generating premature stop codons. All five indels also caused frameshift-generating premature stop codons. The localization of non-silent variants (NSVs) in the HLA genes in the ATL cells are shown in Supplementary Figure 1. All of the NSVs were found in HLA class I genes but not in HLA class II genes. NSVs occurred more often in *HLA-A* and *HLA-B* than in *HLA-C* and localized with the highest frequency to exon 4 (8 mutations out of 17, 47%) of the HLA class I genes.

**Table 1:** A list of somatic mutations identified in 25 ATL cells.

HLA locus	Sample ID	Allele	Mutation			Amino acid substitution*			Ratio of reads**
			Position	ATL	Non-ATL	Position	ATL	Non-ATL	
HLA-A	ATL 08	A*31:01:02:01	1796 (exon 3)	T	C	204	Nonsense	Q	97.3%
	ATL 09	A*02:06:01:01	894 (intron 1)	G/T	G	–	–	–	49.6%
	ATL 13	A*02:06:01:01	2657 (exon 4)	Del	G	290	F (fs*6)	L	94.4%
	ATL 19	A*24:02:01:01	1021 (exon 2)	Ins C	–	26	L (fs*72)	S	98.9%
	ATL 20	A*11:01:01:01	863 (exon 1)	G	T	17	R	L	100%
		A*24:02:01:01	1277 (exon 2)	T	C	111	Nonsense	Q	98.1%
	ATL 23	A*31:01:02:01	2407 (exon 4)	Ins C	–	210	Q (fs*10)	K	90.0%
	ATL 27	A*24:02:01:01	2517 (exon 4)	G/A	G	251	D/N	D	45.6%
			2823 (exon 5)	G/A	G	319	G/R	G	47.8%
HLA-C	ATL 20	C*03:03:01:01	1731 (intron 2, 3′ splice site)	T	A	115	S (fs*3)	R	100%
		C*04:01:01:01	2725 (exon 4)	G	C	250	E	Q	96.4%
HLA-B	ATL 08	B*56:01:01:03	2427 (exon 4)	A	C	207	E	D	82.1%
			2429 (exon 4)	A	C	208	H	P	85.7%
			2555 (exon 4)	T	A	250	L	Q	92.6%
	ATL 09	B*35:01:01:02	2609 (exon 4)	Ins GT	–	268	W/C (fs*29)	W	40.1%
	ATL 11	B*54:01:01:01	1330 (intron 2, 5′ splice site)	A	G	115	D (fs*3)	G	96.8%
	ATL 19	B*55:02:01:03	1646 (exon 3)	T	C	139	Nonsense	Q	98.2%
	ATL 27	B*07:02:01:01	1840–1880 (exon 3-intron 3)	Del 41 bp	–	203	I (fs*35)	L	100%

*Parenthesis indicates that the amino acid numbers from the position at which the frameshift has generated to the position at which the stop codon has newly generated.

**Means ratio of reads that have a mutation in ATL cells.

HLA-LOH was detected by the existence of an allelic imbalance in each HLA loci, as determined by calculating the normalized average depth ratio as the relative depth ratio of ATL cells to non-ATL cells (Supplementary Methods, Supplementary Tables 6 and 7). We found HLA-LOH in ATL cells obtained from 8 patients (Supplementary Fig. 2) and confirmed HLA-LOH by comparing allele sequences of ATL cells to non-ATL cells using the Sanger direct-sequencing (Supplementary Methods and Supplementary Table 8). The analysis strongly supports the notion that HLA-LOH were identified in all HLA-LOH loci, except for HLA-A of ATL01 and HLA-DQB1 of ATL11 (Supplementary Table 9). An overview of the HLA-LOH and NSVs observed in nine ATL patients is shown in Fig. 1A. All of the HLA-LOH and NSVs were found in patients with acute-type ATL, whereas no HLA-LOH or NSVs were detected in patients with chronic-type ATL. Notably, six of the nine patients had HLA-LOH and/or NSVs in both alleles of the same HLA loci.

**Fig. 1:**
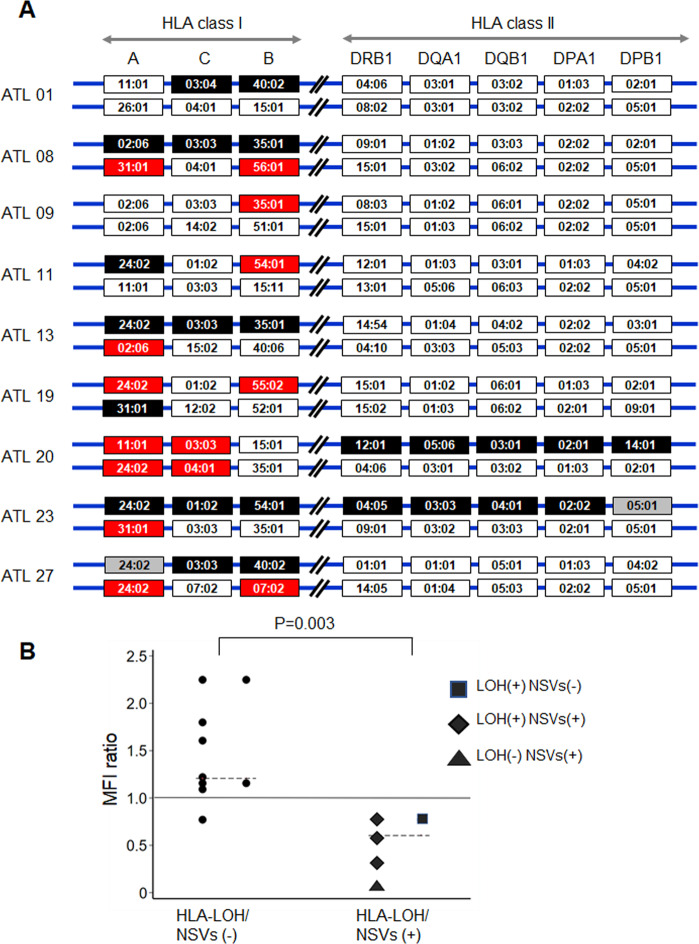
Overview of the HLA gene alterations and HLA class I expression in ATL cells. **A** Overview of the HLA gene alterations found in ATL patients. Nine patients had ATL cells showing a loss of HLA genes and/or non-silent variants (NSVs). Open rectangles indicate intact HLA alleles. Closed black and red rectangles indicate HLA alleles with genetic loss and NSVs, respectively. Closed gray rectangles indicate alleles with suspected but not confirmed genetic loss because of germline homozygosity at the loci. Six of the nine patients had HLA-LOH and/or NSVs in both alleles of the same HLA loci. For example, ATL08 showed loss of the HLA-A*02:06-C*03:03-B*35:01 haplotype and also showed NSVs in HLA-A*31:01 and HLA-B*56:01. ATL 20 had NSVs in both *HLA-A* alleles and in both *HLA-C* alleles. **B** MFI (mean fluorescence intensity) ratios in patients with HLA-LOH and/or NSVs and in those without HLA-LOH and/or NSVs. MFI ratio indicates the relative MFI ratio of ATL cells to non-ATL cells. MFI ratios of HLA class I were compared between patients with and without HLA-LOH/NSVs using the Mann–Whitney *U* test.

We further investigated the surface expression of HLA class I molecules on ATL cells in 14 patients whose PBMC samples were available for flow cytometric analyses. To compare the difference in HLA class I expression between patients with and without HLA-LOH/NSVs, we calculated the mean fluorescence intensity (MFI) ratio of HLA class I (relative MFI ratio of ATL cells to non-ATL cells) in each patient. Representative plots of a patient without HLA-LOH/NSVs (ATL06) and patients with HLA-LOH/NSVs (ATL20 and ATL27) are shown in Supplementary Fig. 3. All patients with HLA-LOH/NSVs had a low MFI in ATL cells compared with that in non-ATL cells. The MFI ratio was significantly lower in patients with HLA-LOH/NSVs than in patients without HLA-LOH/NSVs (Fig. 1B and Supplementary Table 10).

Among patients with acute-type ATL, patients with HLA-LOH/NSVs (*n* = 9) showed a trend for worse survival compared with patients without LOH/NSVs (*n* = 11) (11% vs. 30%, *P* = 0.064), albeit without statistical significance (Supplementary Fig. 4). Because 7 out of 9 patients with HLA-LOH/NSVs had both HLA-LOH and NSVs, it was difficult to analyze the impact on survival separately.

NGS methods for HLA genotyping of full-length HLA genes have emerged, and polymorphisms in the non-coding HLA region detected by full-length HLA typing have been associated with outcomes after allogeneic stem cell transplantation (allo-SCT) [7, 8]. However, full-length HLA allele sequencing has not yet been applied to the detection of HLA-LOH and/or somatic mutations in any cancer cells. A previous integrated genetic study of ATL revealed that less than 10% of patients with ATL had somatic mutations and/or LOH in HLA-B genes [9]. In the present study, eight out of 20 patients (40%) with acute-type ATL had NSVs in HLA class I genes in tumor cells, suggesting that these mutations occur in HLA class I genes with high frequency. Some patients showed a low percentage of mutations in the ATL cells (Table 1), and these mutated cells are presumed to be late-arising subclones [10]. Of the 17 NSVs, two were located in the 5’ and 3’ splice sites in intron 2 and 8 were located in exon 4. Because these mutations are not detected by conventional HLA typing methods, full-length HLA analysis would be useful for the precise detection of HLA gene alterations in tumor cells. Flow cytometric analyses showed reduced HLA class I expression in ATL cells from patients with HLA-LOH/NSVs compared with those from patients without HLA-LOH/NSVs, further supporting the notion that the HLA gene abnormalities occurring in ATL cells would actually lead to downregulation of the cell surface expression of HLA class I molecules.

We also found that HLA-LOH and/or NSVs frequently occurred in both alleles of the same HLA loci, suggesting that both HLA class I alleles would lack the antigen-presenting function to CTLs in patients with HLA-LOH/NSVs. Because most patients with aggressive ATL cannot be cured with conventional chemotherapies [11], allo-SCT is conducted for transplant-eligible patients with newly diagnosed ATL. However, patients with HLA-deficient ATL cells may easily relapse after allo-SCT because of insufficient tumor recognition by HLA-restricted CTLs.

The present study demonstrated that most NSVs were located in *HLA-A* or *HLA-B*, whereas there were only two mutations within the *HLA-C* gene, which is in agreement with the results of a previous report [12]. All HLA-C molecules have an epitope to bind inhibitory killer cell immunoglobulin-like receptors of natural killer (NK) cells [13]. NK cells recognize tumor cells with altered or diminished expression of self-HLA class I molecules. Therefore, it is reasonable to speculate that tumor cells with intact HLA-C may escape NK cell-mediated innate immunity.

Although we could not draw any definitive conclusions due to the small number of patients included, a trend of low survival rate in patients with HLA-LOH/NSVs was observed compared with those without HLA-LOH/NSVs. Further work is required to evaluate the impact of the HLA gene alterations occurring in ATL cells on the clinical outcomes.

In conclusion, SS-SBT–based HLA gene analyses at full-length level revealed that HLA-LOH/NSVs frequently and predominantly occur in HLA class I loci in ATL cells from patients with acute-type ATL. Because allo-HSCT is an essential therapeutic option for aggressive ATL, comprehensive knowledge of HLA gene abnormalities would substantially help to optimize therapeutic modalities.

## Supplementary information

Supplementary Figures

Supplementary Tables

Supplementary methods
