# Supplementary material for: Full-length HLA sequencing in adult T cell leukemia–lymphoma uncovers multiple gene alterations
Source: Leukemia. 2021 Sep 13;35(10):2998–3001. doi: 10.1038/s41375-021-01403-1 (PMC8478651; doi:10.1038/s41375-021-01403-1)
Supplement: Supplementary file 1 — Supplementary Figures [file 41375_2021_1403_MOESM1_ESM.pdf]

Supplementary Figure 1

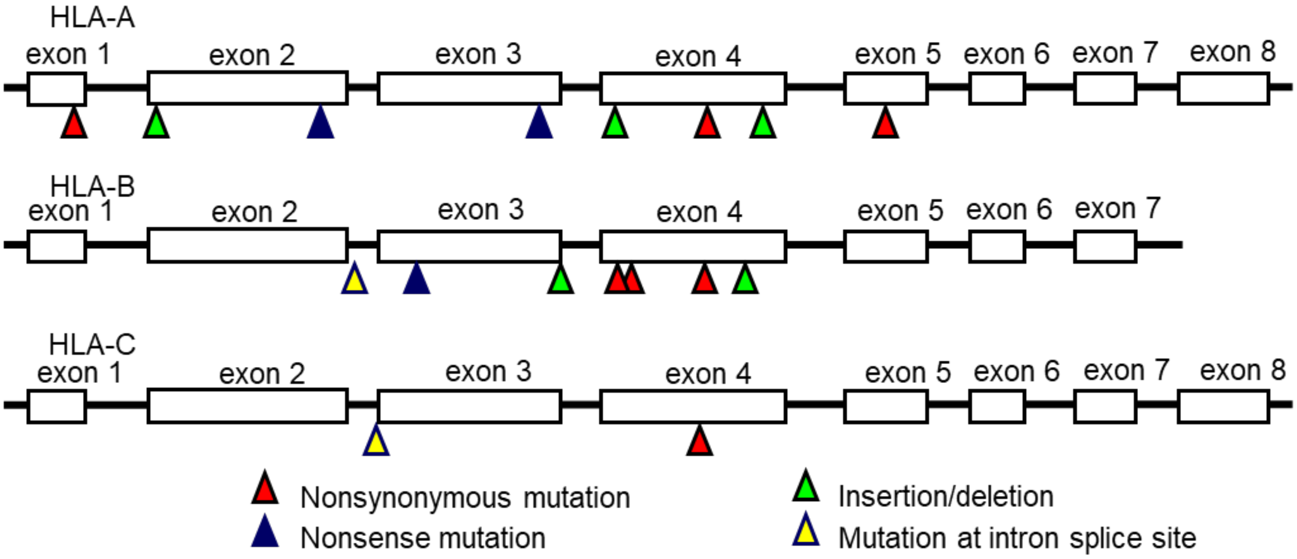

Supplementary Figure 1: Schematic representation of non-silent variants (NSVs) in HLA class I genes. A total of 17 NSVs were detected in HLA class I genes. Triangles indicate the classification of the mutation patterns: nonsynonymous mutation (red), nonsense (blue), insertion/deletion (green), and mutation at an intron splice site (yellow).

Supplementary Figure 2

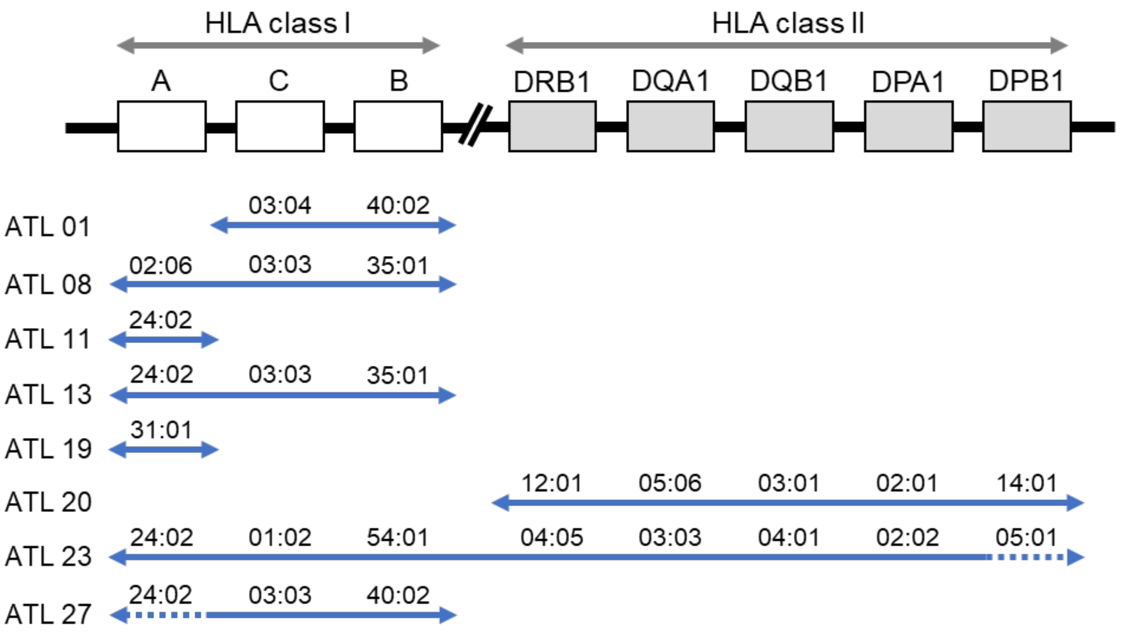

Supplementary Figure 2: Loss of HLA alleles and inferred HLA haplotypes in ATL patients. The arrowed lines indicate regions with genetic loss of the HLA allele and haplotypes in each patient. Loss of HLA-DPB1*05:01 in ATL23 and that of HLA-A*24:02 in ATL27 could not be determined because ATL23 had germline homozygous HLA-DPB1*05:01 alleles and ATL27 had germline homozygous HLA-A*24:02 alleles. Among patients with HLA-LOH, three showed loss of the HLA-A-C-B haplotype (ATL01, 08, and 13) and one showed loss of the HLA-DRB1-DQA1-DQB1-DPA1-DPB1 haplotype (ATL20). ATL cells obtained from ATL23 were speculated to have loss of an extended HLA haplotype from HLA-A to HLA-DPB1. However, we could not determine LOH at the HLA-DPB1 locus because of the patient's germline homozygous HLA-DPB1 allele. Because ATL27 also had germline homozygous HLA-A*24:02, we could not determine whether HLA-LOH occurred in the HLA-A*24:02-C*03:03-B*40:02 haplotype or in the HLA-C*03:03-B*40:02 haplotype.

Supplementary Figure 3

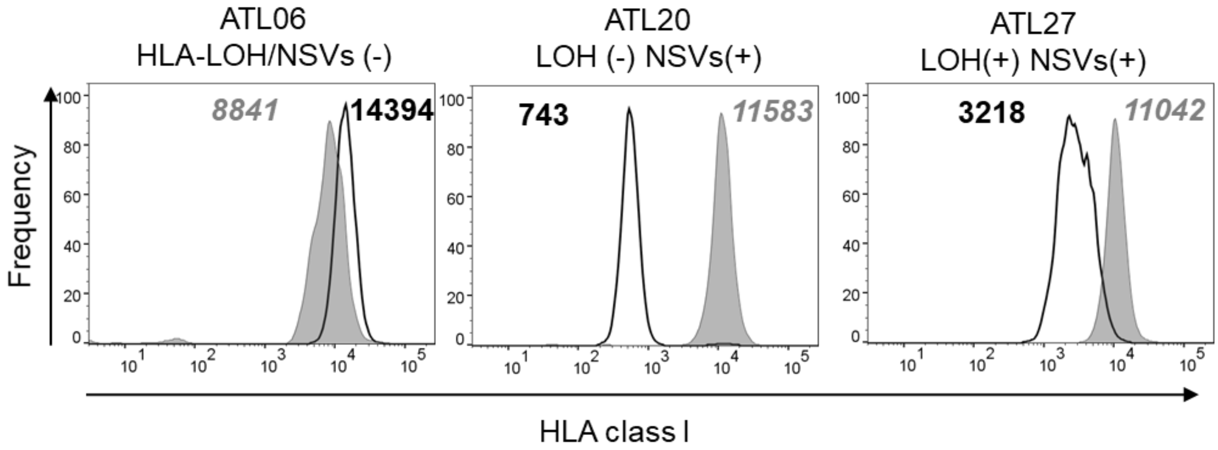

Supplementary Figure 3: Flow cytometric profiles of HLA class I expression in ATL cells (open area) and non-ATL cells (filled area). Representative examples are shown of patients without HLA-LOH and/or NSVs (ATL06) and those with HLA-LOH and/or NSVs (ATL20 and ATL27). The mean fluorescence intensity (MFI) for each result of ATL cells (in bold) or non-ATL cells (in italics) is shown.

Supplementary Figure 4

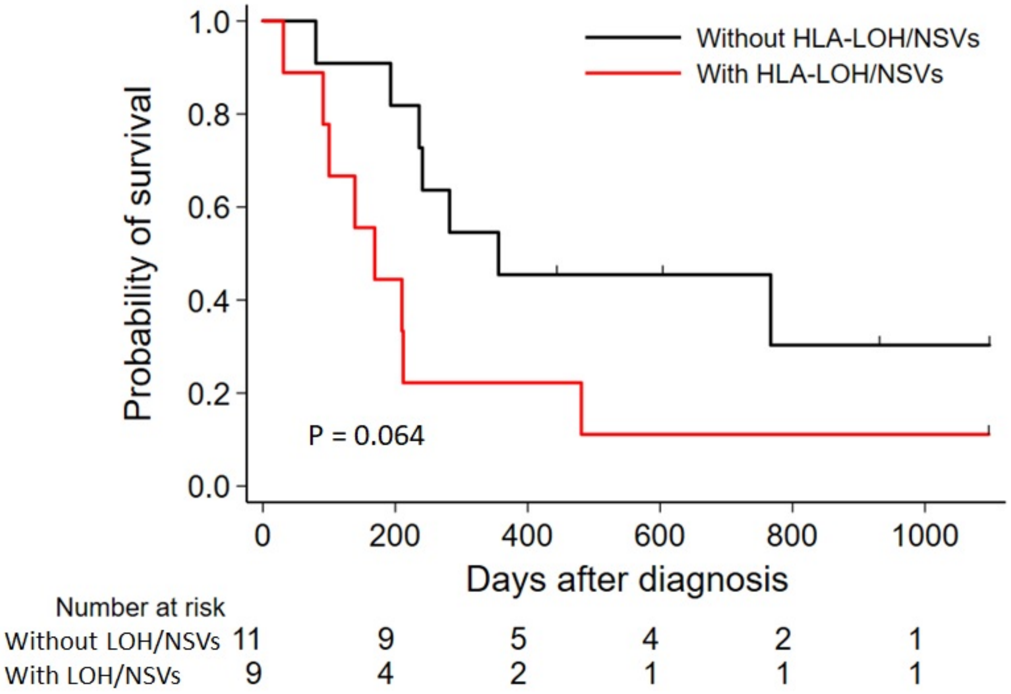

Supplementary Figure 4: Survival curves of acute type ATL patients with or without HLA-LOH/NSVs. Patients with HLA-LOH/NSVs (n = 9) showed a trend for worse survival compared with patients without HLA-LOH/NSVs (n = 11) (P = 0.064).
