# Supplementary material for: Full-length HLA sequencing in adult T cell leukemia–lymphoma uncovers multiple gene alterations
Source: Leukemia. 2021 Sep 13;35(10):2998–3001. doi: 10.1038/s41375-021-01403-1 (PMC8478651; doi:10.1038/s41375-021-01403-1)
Supplement: Supplementary file 2 — Supplementary Tables [file 41375_2021_1403_MOESM2_ESM.pdf]

Supplementary Table 1. Overview of patient characteristics

Characteristics	HLA-LOH/NSVs (-) (N=11)	HLA-LOH/NSVs (+) (N=9)	P value
**Acute type**			
Age (years)			
Median (range)	70 (35-83)	68 (57-80)	0.819
Sex			
Male	3	4	0.642
Female	8	5	
Clinical stage			
I/II	0	0	NA
III/IV	11	9	
ECOG PS			
0/1	8	6	1
> 1	3	3	
Serum albmin (g/dL)			
Median (range)	4.2 (3.0-4.8)	3.3 (2.4-4.5)	0.062
LD (U/L)			
Median (range)	623 (196-3,943)	621 (353-2,313)	0.676
sIL-2R (U/mL)			
Median (range)	16,914 (803-333,344)	32,300 (9,588-178,831)	0.874
Corrected Ca (mmol/L)			
Median (range)	2.57 (2.25-3.97)	2.64 (2.30-3.44)	0.494
Chemotherapy regimen			
CHOP	2	3	0.792
VCAP-AMP-VECP	3	3	
THP-COP	1	1	
Others	5	2	
Stem cell transplantation			
Yes	3	1	0.591
No	8	8	
ATL-PI			
High	3	4	0.723
Intermediate	5	3	
Low	3	2	
JCOG-PI			
High	5	4	
Moderate	6	5	
**Chronic type**			
Age (years)			
Median (range)	74 (54-80)		
Sex			
Male	3		
Female	2		
Clinical stage			
I/II	0		
III/IV	5		
ECOG PS			
0/1	5		
> 1	0		
sIL-2R (U/mL)			
Median (range)	5,542 (4,395-7,128)		

N: Number of patients; ECOG PS: Eastern Cooperative Oncology Group performance status; sIL-2R: soluble interleukin-2 receptor; LD: lactate dehydrogenase; ATL-PI: adult T-cell leukemia/lymphoma prognostic index; JCOG-PI: Japan Clinical Oncology Group prognostic index; NA: not applicable.

Supplementary Table 2. Characteristics of 25 patients analyzed in this study

No.	Age	Sex	Subtype	Survival time from diagnosis (months)	WBC (/μL)	Ly (%)	Ab-Ly (%)	LD (IU/L)	sIL-2R (U/mL)	corrected Ca (mmol/L)	Serum albmin (g/dL)	Simplified ATL-PI or Simplified iATL-PI	Transplantation	Outcome	CADM1+ cells in the ATL cell fraction (%)	CADM1+ cells in the non-ATL cell fraction (%)
ATL01	68	F	acute	7	11800	26	21	621	53449	2.87	2.4	High	No	Dead	99.4	0.2
ATL02	60	F	acute	25	218600	83	3.5	504	68743	2.30	3.3	Intermediate	uBMT	Dead	100	2.3
ATL03	54	F	chronic	9	57600	27.5	60	215	5292	2.40	4.1	Intermediate	No	Dead	97.3	0.8
ATL04	82	M	acute	6	3600	34.5	3	281	3851	2.35	3.9	Intermediate	No	Dead	97.1	2.7
ATL05	70	M	acute	30	10000	47.5	14	204	5777	2.25	3.7	Low	No	Alive	99.8	4.8
ATL06	74	M	chronic	21	17400	3	62	224	4395	2.27	4.1	Intermediate	No	Dead	99.9	0.8
ATL07	73	F	acute	14	16200	9	54.5	623	10222	2.57	4.8	Intermediate	No	Alive	99.7	1.9
ATL08	57	M	acute	4	37000	2.5	74.5	1097	32300	2.45	3.9	Intermediate	No	Dead	99.8	2.8
ATL09	74	F	acute	3	43800	12	58	557	21000	2.64	3.4	High	No	Dead	99.9	0.8
ATL10	65	F	acute	37	127440	3	80	3943	333344	2.99	3.5	Intermediate	No	Alive	99.8	1.7
ATL11	65	F	acute	35	29900	3	85	540	15000	2.37	3.6	Low	No	Alive	99.9	0.7
ATL12	77	F	acute	2	34600	1.5	81.5	414	25900	2.82	3.5	High	No	Dead	99.9	1.1
ATL13	76	F	acute	1	55750	21	59	2313	178831	2.92	2.7	High	No	Dead	99.6	2.6
ATL14	80	F	chronic	87	25300	2	78	214	7128	2.47	NE	High	No	Dead	99.5	0.7
ATL16	83	M	acute	7	38000	5.5	75.5	1119	73600	3.97	3.6	High	No	Dead	99.8	1.1
ATL17	67	M	chronic	31	19200	4	80.5	226	5542	2.40	4.8	Intermediate	No	Alive	99.6	1.7
ATL18	66	F	acute	7	7800	13	29	945	46744	3.59	3.0	High	No	Dead	99.8	0.7
ATL19	68	M	acute	5	6200	14.5	5.5	354	9588	3.44	2.7	Intermediate	No	Dead	99.6	1.6
ATL20	80	M	acute	2	10500	4	16.5	1116	75000	3.17	3.2	High	No	Dead	99.9	2.9
ATL21	35	F	acute	19	5700	15	21.5	860	14413	2.35	4.4	Low	rPBSCT	Alive	99.7	8
ATL22	78	M	chronic	27	8500	23	36	283	5884	2.40	4.3	Intermediate	No	Dead	99.8	7.4
ATL23	65	M	acute	6	127700	18.5	72	633	41559	2.37	3.3	Intermediate	No	Dead	99.8	0.5
ATL24	74	F	acute	11	7900	26.5	10	196	803	2.25	4.2	Intermediate	No	Dead	99.2	0.4
ATL26	56	F	acute	9	55900	3.5	79.5	739	16914	2.59	4.5	Low	uBMT	Dead	99.9	1.1
ATL27	62	F	acute	15	6200	20	51.5	353	15100	2.30	4.5	Low	uBMT	Dead	99.7	0

ATL-PI: adult T-cell leukemia/lymphoma prognositic index; iATL-PI: indolent adult T-cell leukemia/lymphoma prognostic index; LOH: loss of heterozygosity; uBMT: unrelated bone marrow transplantation; rPBSCT:related peripheral blood stem cell transplantation; NE: not examined.

Supplementary Table 3. Sequence read information obtained by the Ion S5 system

A. ATL cells

DNA sample ID	Disease type	Draft read numbers	Draft read bases (b)	Average read length (b)	Mode read length (b)	Average quality value
ATL01	Acute	685,372	220,044,807	321.1	411	27.3
ATL02	Acute	760,874	222,986,839	293.1	363	27.7
ATL03	Chronic	540,029	165,203,166	305.9	382	27.3
ATL04	Acute	766,376	245,475,265	320.3	421	27.1
ATL05	Acute	785,516	257,929,711	328.4	419	27.0
ATL06	Chronic	682,261	207,547,478	304.2	403	27.6
ATL07	Acute	609,425	180,319,924	295.9	352	28.2
ATL08	Acute	1,110,814	337,543,918	303.9	368	27.9
ATL09	Acute	1,129,716	341,318,543	302.1	380	27.9
ATL10	Acute	1,103,000	334,491,610	303.3	368	28.0
ATL11	Acute	1,271,677	380,190,688	299.0	358	28.0
ATL12	Acute	1,090,571	318,766,480	292.3	332	28.4
ATL13	Acute	761,027	232,055,853	304.9	412	27.5
ATL14	Chronic	607,129	201,622,449	332.1	431	26.8
ATL16	Acute	519,441	168,455,512	324.3	443	26.6
ATL17	Chronic	430,771	142,598,550	331.0	446	26.3
ATL18	Acute	379,186	125,027,645	329.7	444	26.2
ATL19	Acute	899,150	263,881,556	293.5	368	28.4
ATL20	Acute	744,029	221,585,324	297.8	386	28.0
ATL21	Acute	842,582	254,211,770	301.7	383	28.0
ATL22	Chronic	707,155	207,789,826	293.8	380	28.0
ATL23	Acute	812,834	249,563,701	307.0	384	27.8
ATL24	Acute	945,697	290,036,630	306.7	358	28.2
ATL26	Acute	528,466	173,680,643	328.7	430	26.8
ATL27	Acute	301,628	101,453,443	336.4	430	26.8
Average		760,589	233,751,253	310.3	394	27.5
±SD		249,727	71,454,116	14.5	32.7	0.6
Maximum value		1,271,677	380,190,688	336.4	446	28.4
Minimum value		301,628	101,453,443	292.3	332	26.2

B. Non-ATL cells

DNA sample ID	Disease type	Draft read numbers	Draft read bases (b)	Average read length (b)	Mode read length (b)	Average quality value
NATL01	Acute	549,187	169,871,198	309.3	339	27.9
NATL02	Acute	992,945	317,347,355	319.6	408	27.7
NATL03	Chronic	737,670	235,441,552	319.2	411	27.5
NATL04	Acute	803,588	258,169,077	321.3	368	27.6
NATL05	Acute	1,017,205	339,215,419	333.5	421	27.4
NATL06	Chronic	572,695	187,384,678	327.2	410	27.6
NATL07	Acute	937,257	295,472,347	315.3	395	27.9
NATL08	Acute	895,753	281,253,527	314.0	361	27.7
NATL09	Acute	719,477	231,816,180	322.2	362	27.5
NATL10	Acute	1,065,080	347,578,640	326.3	423	27.6
NATL11	Acute	2,950,079	990,121,527	335.6	421	26.8
NATL12	Acute	796,251	261,469,661	328.4	400	27.7
NATL13	Acute	815,771	270,534,199	331.6	403	27.5
NATL14	Chronic	695,870	225,702,877	324.3	421	27.5
NATL16	Acute	844,165	278,240,627	329.6	426	27.3
NATL17	Chronic	708,741	222,881,812	314.5	412	27.6
NATL18	Acute	1,108,172	347,296,555	313.4	395	27.7
NATL19	Acute	843,442	265,520,816	314.8	390	27.8
NATL20	Acute	636,972	195,079,018	306.3	363	27.6
NATL21	Acute	1,006,302	313,283,780	311.3	371	27.5
NATL22	Chronic	615,860	196,681,668	319.4	385	27.3
NATL23	Acute	618,962	194,909,814	314.9	382	27.4
NATL24	Acute	588,847	188,043,133	319.3	372	27.5
NATL26	Acute	614,130	197,440,587	321.5	353	27.5
NATL27	Acute	729,273	235,870,960	323.4	417	27.5
Average		874,548	281,865,080	320.6	392.4	27.5
±SD		462,319	156,855,931	7.7	25.3	0.2
Maximum value		2,950,079	990,121,527	335.6	426	27.9
Minimum value		549,187	169,871,198	306.3	339	26.8

Supplementary Table 4: HLA typing results assigned in 25 ATL and/or non-ATL cells

Sample ID	HLA-A		HLA-C		HLA-B		HLA-DRB1	
NATL01	A*11:01:01:01	A*26:01:01:01	C*03:04:01:02	C*04:01:01:01	B*15:01:01:01	B*40:02:01:01	DRB1*04:06:01	DRB1*08:02:01:01
NATL02	A*02:01:01:01	A*02:06:01:01	C*03:03:01:01	C*14:02:01:01	B*35:01:01:51	B*51:01:01:01	DRB1*09:01:02:01	DRB1*15:01:01:03
NATL03	A*02:01:01:01	A*31:01:02:01	C*03:03:01:01	C*15:02:01:01	B*15:11:01	B*40:01:02:01/04	DRB1*09:01:02:01	DRB1*11:01:01:04
NATL04	A*02:06:01:01	A*31:01:02:01	C*03:04:01:02	C*14:02:01:01	B*40:01:02:01/04	B*51:01:01:01	DRB1*09:01:02:01	DRB1*14:05:01:02
NATL05	A*02:01:01:01	A*26:01:01:01	C*03:03:01:01	C*03:04:01:02	B*35:01:01:02	B*40:02:01:01	DRB1*08:02:01:01	DRB1*15:01:01:03
NATL06	A*02:01:01:01	A*02:06:01:01	C*03:04:01:02	C*14:02:01:01	B*40:02:01:01	B*51:01:01:01	DRB1*04:05:01:01	DRB1*09:01:02:01
NATL07	A*02:01:01:01	A*24:02:01:01	C*01:02:01:05	C*15:02:01:01	B*51:01:01:01	B*54:01:01:01	DRB1*14:05:01:02	DRB1*15:01:01:03
NATL08	A*02:06:01:01	A*31:01:02:01	C*03:03:01:01	C*04:01:01:01	B*35:01:01:02	B*56:01:01:03	DRB1*09:01:02:01	DRB1*15:01:01:03
NATL09	A*02:06:01:01	-	C*03:03:01:01	C*14:02:01:01	B*35:01:01:51	B*51:01:01:01	DRB1*08:03:02:02	DRB1*15:01:01:03
NATL10	A*02:01:01:01	A*24:02:01:01	C*03:03:01:01	C*07:02:01:15	B*35:01:01:51	B*40:01:02:01/04	DRB1*09:01:02:01	DRB1*15:01:01:03
NATL11	A*11:01:01:01	A*24:02:01:01	C*01:02:01:05	C*03:03:01:01	B*15:11:01	B*54:01:01:01	DRB1*12:01:01:04	DRB1*13:01:01:01
NATL12	A*02:06:01:01	A*24:02:01:01	C*01:02:01:01	C*03:04:01:02	B*40:01:02:01/04	B*59:01:01:01	DRB1*04:05:01:01	DRB1*08:03:02:02
NATL13	A*02:06:01:01	A*24:02:01:01	C*03:03:01:01	C*15:02:01:01	B*35:01:01:02	B*40:06:01:01	DRB1*04:10:03	DRB1*14:54:01:08
NATL14	A*02:01:01:01	A*24:02:01:01	C*03:03:01:01	-	B*35:01:01:02	B*40:02:01:01	DRB1*08:02:01:01	DRB1*15:01:01:03
NATL16	A*24:02:01:01	-	C*03:03:01:01	C*03:04:01:02	B*51:01:01:05	B*55:02:01:03	DRB1*04:10:01	DRB1*08:02:01:01
NATL17	A*02:01:01:01	A*24:02:01:01	C*03:04:01:02	C*04:01:01:01	B*40:06:01:01	B*56:01:01:03	DRB1*04:05:01:01	-
NATL18	A*02:06:01:01	A*11:01:01:01	C*04:01:01:01	C*07:02:01:108	B*15:01:01:01	-	DRB1*04:06:01	DRB1*15:01:01:03
NATL19	A*24:02:01:01	A*31:01:02:01	C*01:02:01:01	C*12:02:02:01	B*52:01:01:02	B*55:02:01:03	DRB1*15:01:01:03	DRB1*15:02:01:03
NATL20	A*11:01:01:01	A*24:02:01:01	C*03:03:01:01	C*04:01:01:01	B*15:01:01:01	B*35:01:03	DRB1*04:06:01	DRB1*12:01:01:04
NATL21	A*24:02:01:01	A*26:01:01:01	C*08:03:01	C*14:02:01:01	B*48:01:01:01	B*51:01:01:01	DRB1*14:54:01:06	DRB1*15:01:01:03
NATL22	A*26:03:01:01	A*31:01:02:01	C*03:03:01:01	C*03:04:01:02	B*15:01:01:01	B*35:01:01:51	DRB1*15:01:01:03	-
NATL23	A*24:02:01:01	A*31:01:02:01	C*01:02:01:05	C*03:03:01:01	B*35:01:01:02	B*54:01:01:01	DRB1*09:01:02:02	DRB1*04:05:01:01
NATL24	A*11:01:01:01	A*24:02:01:01	C*01:02:01:05	C*07:02:01:03	B*07:02:01:01	B*54:01:01:01	DRB1*01:01:01:01	DRB1*04:05:01:01
NATL26	A*11:01:01:01	A*24:02:01:01	C*01:02:01:05	C*15:02:01:01	B*40:06:01:01	B*54:01:01:01	DRB1*04:05:01:01	DRB1*09:01:02:01
NATL27	A*24:02:01:01	-	C*03:03:01:01	C*07:02:01:03	B*07:02:01:01	B*40:02:01:01	DRB1*01:01:01:01	DRB1*14:05:01:02

	HLA-DQA1		HLA-DQB1		HLA-DPA1		HLA-DPB1	
NATL01	DQA1*03:01:01:01	-	DQB1*03:02:01:01	-	DPA1*01:03:01:01	DPA1*02:02:02:02	DPB1*02:01:02:01	DPB1*05:01:01:01
NATL02	DQA1*01:02:01:01	DQA1*03:02:01:01	DQB1*03:01:01:01	DQB1*06:02:01:01	DPA1*02:01:01:01	DPA1*02:02:02:01	DPB1*05:01:01:01	DPB1*13:01:01:06
NATL03	DQA1*03:02:01:01	DQA1*05:05:01:09	DQB1*03:01:01:03	DQB1*03:03:02:02/03	DPA1*01:03:01:01	DPA1*02:02:02:01	DPB1*02:01:02:48	DPB1*05:01:01:05
NATL04	DQA1*01:04:01:01	DQA1*03:02:01:01	DQB1*03:03:02:02/03	DQB1*05:03:01:02	DPA1*01:03:01:01	DPA1*01:03:01:08	DPB1*02:01:02:11	DPB1*02:01:02:32
NATL05	DQA1*01:02:01:01	DQA1*03:01:01:01	DQB1*03:02:01:01	DQB1*06:02:01:01	DPA1*01:03:01:01	DPA1*02:02:02:01	DPB1*02:01:02:01	DPB1*05:01:01:01
NATL06	DQA1*03:02:01:01	DQA1*03:03:01:03	DQB1*03:03:02:02/03	DQB1*04:01:01:02	DPA1*02:02:02:01	-	DPB1*05:01:01:01	DPB1*05:01:01:08
NATL07	DQA1*01:02:01:01	DQA1*01:04:01:01	DQB1*05:03:01:02	DQB1*06:02:01:01	DPA1*02:02:02:01	-	DPB1*05:01:01:01	DPB1*05:01:01:18
NATL08	DQA1*01:02:01:01	DQA1*03:02:01:01	DQB1*03:03:02:02/03	DQB1*06:02:01:01	DPA1*02:02:02:01	DPA1*02:02:02:11	DPB1*02:01:02:29	DPB1*05:01:01:01
NATL09	DQA1*01:02:01:01	DQA1*01:03:01:01	DQB1*06:01:01:01	DQB1*06:02:01:01	DPA1*02:02:02:01	-	DPB1*05:01:01:01	DPB1*05:01:01:05
NATL10	DQA1*01:02:01:01	DQA1*03:02:01:01	DQB1*03:03:02:02/03	DQB1*06:02:01:01	DPA1*02:02:02:01	-	DPB1*02:01:02:29	DPB1*05:01:01:01
NATL11	DQA1*01:03:01:12	DQA1*05:06:01:03	DQB1*03:01:01:01	DQB1*06:03:01:01	DPA1*01:03:01:05	DPA1*02:02:02:01	DPB1*04:02:01:02	DPB1*05:01:01:01
NATL12	DQA1*01:03:01:07	DQA1*03:03:01:03	DQB1*04:01:01:02	DQB1*06:01:01:01	DPA1*01:03:01:01	DPA1*01:03:01:05	DPB1*02:01:02:01	DPB1*04:02:01:02
NATL13	DQA1*01:04:01:01	DQA1*03:03:01:02	DQB1*04:02:01:05	DQB1*05:03:01:04	DPA1*02:02:02:01	DPA1*02:02:02:02	DPB1*03:01:01:08	DPB1*05:01:01:01
NATL14	DQA1*01:02:01:01	DQA1*04:01:01:01	DQB1*04:02:01:04	DQB1*06:02:01:01	DPA1*01:03:01:08	DPA1*02:02:02:01	DPB1*02:01:02:01	DPB1*02:01:02:32
NATL16	DQA1*03:01:01:01	DQA1*03:03:01:01	DQB1*03:02:01:01	DQB1*04:02:01:05	DPA1*01:03:01:01	DPA1*02:02:02:01	DPB1*03:01:01:09	DPB1*47:01:01:01

NATL17	DQA1*03:03:01:03	-	DQB1*04:01:01:02	-	DPA1*01:03:01:08	DPA1*02:02:02:01	DPB1*02:01:02:32	DPB1*05:01:01:01
NATL18	DQA1*01:02:01:01	DQA1*03:01:01:01	DQB1*03:02:01:01	DQB1*06:02:01:01	DPA1*01:03:01:01	-	DPB1*02:01:02:01	-
NATL19	DQA1*01:02:01:01	DQA1*01:03:01:01	DQB1*06:01:01:01	DQB1*06:02:01:01	DPA1*01:03:01:01	DPA1*02:01:01:02	DPB1*02:01:02:01	DPB1*09:01:01
NATL20	DQA1*03:01:01:01	DQA1*05:06:01:01	DQB1*03:02:01:01	DQB1*03:01:01:01	DPA1*01:03:01:01	DPA1*02:01:01:02	DPB1*02:01:02:01	DPB1*14:01:01:01
NATL21	DQA1*01:02:01:01	DQA1*01:04:01:01	DQB1*05:02:01:03	DQB1*06:02:01:01	DPA1*01:03:01:01	DPA1*01:03:01:05	DPB1*02:01:02:01	DPB1*04:02:01:02
NATL22	DQA1*01:02:01:01	-	DQB1*06:02:01:01	-	DPA1*02:01:01:01	DPA1*02:02:02:01	DPB1*02:01:02:29	DPB1*13:01:01:06
NATL23	DQA1*03:02:01:01	DQA1*03:03:01:03	DQB1*03:03:02:02/03	DQB1*04:01:01:02	DPA1*02:01:01:13	DPA1*02:02:02:01	DPB1*05:01:01:01	-
NATL24	DQA1*01:01:01:04	DQA1*03:03:01:03	DQB1*04:01:01:02	DQB1*05:01:01:03	DPA1*01:03:01:05	-	DPB1*04:02:01:02	-
NATL26	DQA1*03:02:01:01	DQA1*03:03:01:03	DQB1*03:03:02:02/03	DQB1*04:01:01:02	DPA1*02:02:02:01	-	DPB1*05:01:01:01	DPB1*05:01:01:05
NATL27	DQA1*01:01:01:04	DQA1*01:04:01:01	DQB1*05:01:01:03	DQB1*05:03:01:01	DPA1*01:03:01:05	DPA1*02:02:02:02	DPB1*04:02:01:02	DPB1*05:01:01:01

*HLA alleles shown by red letter indicate novel alleles identified in both of ATL and non-ATL cells. Detail information of the novel alleles were indicated to Supplementary Table 5.

Supplementary Table 5. A list for novel HLA alleles in 25 ATL and non-ATL cells

HLA locus	Sample ID	Allele	Mutation			Amino acid substitution			Ratio of reads**
			Position	ATL	Non-ATL	Position	ATL	Non-ATL	
HLA-C	ATL 18	C*07:02:01:108	1136 (intron 1)	G	G	-	-	-	100%
HLA-B	ATL 02	B*35:01:01:51	3837 (3`UTR)	T	T	-	-	-	100%
	ATL 09	B*35:01:01:51	3837 (3`UTR)	T	T	-	-	-	100%
	ATL 10	B*35:01:01:51	3837 (3`UTR)	T	T	-	-	-	100%
	ATL 22	B*35:01:01:51	3837 (3`UTR)	T	T	-	-	-	100%
HLA-DRB1	ATL 13	DRB1*14:54:01:08	11169 (intron 2)	A	A	-	-	-	99.8%
	ATL 21	DRB1*14:54:01:06	3631 (intron 1)	G	G	-	-	-	99.7%
			10987 (intron 2)	A	A	-	-	-	99.3%
			11169 (intron 2)	A	A	-	-	-	99.3%
HLA-DQA1	ATL 11	DQA1*01:03:01:12	3453 (intron 1)	C	C	-	-	-	100%
	ATL 11	DQA1*05:06:01:03	2606 (intron 1)	G	G	-	-	-	100%
HLA-DQB1	ATL 13	DQB1*05:03:01:04	6303 (intron 3)	G	G	-	-	-	98.8%
HLA-DPA1	ATL 08	DPA1*02:02:02:11	5362 (intron 4)	G/A	G/A	-	-	-	42.4%
	ATL 23	DPA1*02:01:01:13	500-2078 (intron 1)	SNV 17 bp<br>Ins 10 bp	SNV 17 bp<br>Ins 10 bp	-	-	-	98.3%
HLA-DPB1	ATL 03	DPB1*02:01:02:48	7246 (intron 2)	Del	Del	-	-	-	98.6%
	ATL 07	DPB1*05:01:01:18	11408 (3'UTR)	G/A	G/A	-	-	-	54.7%

*means ratio of reads that have mutation in ATL cells.

Supplementary Table 6. Calculation of relative depth ratios of ATL to non-ATL cells in HLA genes

Sample ID	HLA-A Allele name Allele1	HLA-A Allele name Allele2	HLA-A Read number Allele1	HLA-A Read number Allele2	HLA-A Allele 1 /Allele 2	HLA-A NR*
NATL01	A*11:01:01:01	A*26:01:01:01	5,645	6,753	0.84	1
ATL01	A*11:01:01:01	A*26:01:01:01	1,388	9,886	0.14	0.17
NATL02	A*02:01:01:01	A*02:06:01:01	158	135	1.17	1
ATL02	A*02:01:01:01	A*02:06:01:01	104	97	1.07	0.92
NATL03	A*02:01:01:01	A*31:01:02:01	8,676	8,980	0.97	1
ATL03	A*02:01:01:01	A*31:01:02:01	5,894	5,527	1.07	0.91
NATL04	A*02:06:01:01	A*31:01:02:01	8,556	8,498	1.01	1
ATL04	A*02:06:01:01	A*31:01:02:01	10,821	10,511	1.03	0.98
NATL05	A*02:01:01:01	A*26:01:01:01	11,957	10,383	1.15	1
ATL05	A*02:01:01:01	A*26:01:01:01	11,561	10,284	1.12	0.98
NATL06	A*02:01:01:01	A*02:06:01:01	167	181	0.92	1
ATL06	A*02:01:01:01	A*02:06:01:01	125	131	0.95	0.97
NATL07	A*02:01:01:01	A*24:02:01:01	12,909	12,718	1.02	1
ATL07	A*02:01:01:01	A*24:02:01:01	7,738	7,193	1.08	0.94
NATL08	A*02:06:01:01	A*31:01:02:01	12,759	11,873	1.07	1
ATL08	A*02:06:01:01	A*31:01:02:01	233	18,861	0.01	0.01
NATL09	A*02:06:01:01	-	-	-	-	-
ATL09	A*02:06:01:01	-	-	-	-	-
NATL10	A*02:01:01:01	A*24:02:01:01	12,593	11,814	1.07	1
ATL10	A*02:01:01:01	A*24:02:01:01	9,148	8,904	1.03	0.96
NATL11	A*11:01:01:01	A*24:02:01:01	6,103	7,693	0.79	1
ATL11	A*11:01:01:01	A*24:02:01:01	26,772	386	69.36	0.01
NATL12	A*02:06:01:01	A*24:02:01:01	9,693	9,124	1.06	1
ATL12	A*02:06:01:01	A*24:02:01:01	9,953	9,350	1.06	1.00
NATL13	A*02:06:01:01	A*24:02:01:01	8,669	8,472	1.02	1
ATL13	A*02:06:01:01	A*24:02:01:01	10,996	134	82.06	0.01
NATL14	A*02:01:01:01	A*24:02:01:01	3,880	3,649	1.06	1
ATL14	A*02:01:01:01	A*24:02:01:01	4,530	4,032	1.12	0.95
NATL16	A*24:02:01:01	-	-	-	-	-
ATL16	A*24:02:01:01	-	-	-	-	-
NATL17	A*02:01:01:01	A*24:02:01:01	6,456	6,451	1.00	1
ATL17	A*02:01:01:01	A*24:02:01:01	4,244	3,829	1.11	0.90
NATL18	A*02:06:01:01	A*11:01:01:01	7,748	5,648	1.37	1
ATL18	A*02:06:01:01	A*11:01:01:01	3,242	2,386	1.36	0.99
NATL19	A*24:02:01:01	A*31:01:02:01	8,542	8,223	1.04	1
ATL19	A*24:02:01:01	A*31:01:02:01	13,143	422	31.14	0.03
NATL20	A*11:01:01:01	A*24:02:01:01	3,503	4,565	0.77	1
ATL20	A*11:01:01:01	A*24:02:01:01	5,114	5,698	0.90	0.85
NATL21	A*24:02:01:01	A*26:01:01:01	7,071	6,877	1.03	1
ATL21	A*24:02:01:01	A*26:01:01:01	5,997	5,571	1.08	0.96
NATL22	A*26:03:01:01	A*31:01:02:01	4,488	4,236	1.06	1
ATL22	A*26:03:01:01	A*31:01:02:01	5,615	5,912	0.95	0.90
NATL23	A*24:02:01:01	A*31:01:02:01	4,065	4,465	0.91	1
ATL23	A*24:02:01:01	A*31:01:02:01	356	12,492	0.03	0.03
NATL24	A*11:01:01:01	A*24:02:01:01	3,090	3,511	0.88	1
ATL24	A*11:01:01:01	A*24:02:01:01	7,881	9,779	0.81	0.92
NATL26	A*11:01:01:01	A*24:02:01:01	3,506	5,055	0.69	1
ATL26	A*11:01:01:01	A*24:02:01:01	3,824	4,168	0.92	0.76
NATL27	A*24:02:01:01	-	-	-	-	-
ATL27	A*24:02:01:01	-	-	-	-	-

Sample ID	HLA-C Allele name Allele1	HLA-C Allele name Allele2	HLA-C Read number Allele1	HLA-C Read number Allele2	HLA-C Allele 1 /Allele 2	HLA-C NR*
NATL01	C*03:04:01:02	C*04:01:01:01	5,420	5,360	1.01	1
ATL01	C*03:04:01:02	C*04:01:01:01	1,296	8,790	0.15	0.15
NATL02	C*03:03:01:01	C*14:02:01:01	8,845	6,882	1.29	1
ATL02	C*03:03:01:01	C*14:02:01:01	6,312	5,632	1.12	0.87
NATL03	C*03:03:01:01	C*15:02:01:01	6,778	4,436	1.53	1
ATL03	C*03:03:01:01	C*15:02:01:01	5,739	4,167	1.38	0.90
NATL04	C*03:04:01:02	C*14:02:01:01	6,007	5,382	1.12	1
ATL04	C*03:04:01:02	C*14:02:01:01	6,482	7,167	0.90	0.81
NATL05	C*03:03:01:01	C*03:04:01:02	87	67	1.30	1
ATL05	C*03:03:01:01	C*03:04:01:02	154	95	1.62	0.80
NATL06	C*03:04:01:02	C*14:02:01:01	4,918	4,017	1.22	1
ATL06	C*03:04:01:02	C*14:02:01:01	6,962	6,280	1.11	0.91
NATL07	C*01:02:01:05	C*15:02:01:01	6,796	5,902	1.15	1
ATL07	C*01:02:01:05	C*15:02:01:01	3,753	3,260	1.15	1.00
NATL08	C*03:03:01:01	C*04:01:01:01	12,544	9,870	1.27	1
ATL08	C*03:03:01:01	C*04:01:01:01	252	16,472	0.02	0.01
NATL09	C*03:03:01:01	C*14:02:01:01	4,634	3,209	1.44	1
ATL09	C*03:03:01:01	C*14:02:01:01	7,290	6,087	1.20	0.83
NATL10	C*03:03:01:01	C*07:02:01:15	12,491	9,263	1.35	1
ATL10	C*03:03:01:01	C*07:02:01:15	8,770	6,613	1.33	0.98
NATL11	C*01:02:01:05	C*03:03:01:01	15,933	19,481	0.82	1
ATL11	C*01:02:01:05	C*03:03:01:01	22,401	17,981	1.25	0.66
NATL12	C*01:02:01:01	C*03:04:01:02	7,353	8,196	0.90	1
ATL12	C*01:02:01:01	C*03:04:01:02	6,683	7,009	0.95	0.94
NATL13	C*03:03:01:01	C*15:02:01:01	6,634	5,174	1.28	1
ATL13	C*03:03:01:01	C*15:02:01:01	174	7,625	0.02	0.02
NATL14	C*03:03:01:01	-	-	-	-	-
ATL14	C*03:03:01:01	-	-	-	-	-
NATL16	C*03:03:01:01	C*03:04:01:02	63	46	1.37	1
ATL16	C*03:03:01:01	C*03:04:01:02	31	24	1.29	0.94
NATL17	C*03:04:01:02	C*04:01:01:01	5,327	4,762	1.12	1
ATL17	C*03:04:01:02	C*04:01:01:01	2,869	2,723	1.05	0.94
NATL18	C*04:01:01:01	C*07:02:01:108**	5,365	5,490	0.98	1
ATL18	C*04:01:01:01	C*07:02:01:108**	2,731	2,361	1.16	0.84
NATL19	C*01:02:01:01	C*12:02:02:01	5,991	6,296	0.95	1
ATL19	C*01:02:01:01	C*12:02:02:01	4,706	4,682	1.01	0.95
NATL20	C*03:03:01:01	C*04:01:01:01	4,190	3,172	1.32	1
ATL20	C*03:03:01:01	C*04:01:01:01	4,854	4,257	1.14	0.86
NATL21	C*08:03:01	C*14:02:01:01	6,911	5,261	1.31	1
ATL21	C*08:03:01	C*14:02:01:01	5,277	4,262	1.24	0.94
NATL22	C*03:03:01:01	C*03:04:01:02	31	24	1.29	1
ATL22	C*03:03:01:01	C*03:04:01:02	69	49	1.41	0.92
NATL23	C*01:02:01:05	C*03:03:01:01	2,751	3,457	0.80	1
ATL23	C*01:02:01:05	C*03:03:01:01	222	8,304	0.03	0.03
NATL24	C*01:02:01:05	C*07:02:01:03	3,847	3,969	0.97	1
ATL24	C*01:02:01:05	C*07:02:01:03	6,701	7,123	0.94	0.97
NATL26	C*01:02:01:05	C*15:02:01:01	3,063	2,065	1.48	1
ATL26	C*01:02:01:05	C*15:02:01:01	2,878	2,200	1.31	0.88
NATL27	C*03:03:01:01	C*07:02:01:03	6,596	5,324	1.24	1
ATL27	C*03:03:01:01	C*07:02:01:03	110	3,213	0.03	0.03

Sample ID	HLA-B Allele name Allele1	HLA-B Allele name Allele2	HLA-B Read number Allele1	HLA-B Read number Allele2	HLA-B Allele 1 /Allele 2	HLA-B NR*
NATL01	B*15:01:01:01	B*40:02:01:01	5,699	5,258	1.08	1
ATL01	B*15:01:01:01	B*40:02:01:01	7,493	1,012	7.40	0.15
NATL02	B*35:01:01:51**	B*51:01:01:01	785	881	0.89	1
ATL02	B*35:01:01:51**	B*51:01:01:01	648	777	0.83	0.94
NATL03	B*15:11:01	B*40:01:02:01/04	5,708	6,730	0.85	1
ATL03	B*15:11:01	B*40:01:02:01/04	4,293	4,434	0.97	0.88
NATL04	B*40:01:02:01/04	B*51:01:01:01	5,353	6,199	0.86	1
ATL04	B*40:01:02:01/04	B*51:01:01:01	7,726	7,666	1.01	0.86
NATL05	B*35:01:01:02	B*40:02:01:01	7,067	7,034	1.00	1
ATL05	B*35:01:01:02	B*40:02:01:01	6,713	6,938	0.97	0.96
NATL06	B*40:02:01:01	B*51:01:01:01	3,392	3,837	0.88	1
ATL06	B*40:02:01:01	B*51:01:01:01	5,537	6,247	0.89	1.00
NATL07	B*51:01:01:01	B*54:01:01:01	6,742	6,218	1.08	1
ATL07	B*51:01:01:01	B*54:01:01:01	3,153	2,877	1.10	0.99
NATL08	B*35:01:01:02	B*56:01:01:03	6,971	6,553	1.06	1
ATL08	B*35:01:01:02	B*56:01:01:03	134	5,127	0.03	0.02
NATL09	B*35:01:01:51**	B*51:01:01:01	494	489	1.01	1
ATL09	B*35:01:01:51**	B*51:01:01:01	650	692	0.94	0.93
NATL10	B*35:01:01:51**	B*40:01:02:01/04	8,189	9,732	0.84	1
ATL10	B*35:01:01:51**	B*40:01:02:01/04	6,150	7,240	0.85	0.99
NATL11	B*15:11:01	B*54:01:01:01	6,042	5,130	1.18	1
ATL11	B*15:11:01	B*54:01:01:01	10,505	12,564	0.84	0.71
NATL12	B*40:01:02:01/04	B*59:01:01:01	9,682	7,320	1.32	1
ATL12	B*40:01:02:01/04	B*59:01:01:01	7,964	5,905	1.35	0.98
NATL13	B*35:01:01:02	B*40:06:01:01	5,260	4,503	1.17	1
ATL13	B*35:01:01:02	B*40:06:01:01	52	7,045	0.01	0.01
NATL14	B*35:01:01:02	B*40:02:01:01	2,528	2,676	0.94	1
ATL14	B*35:01:01:02	B*40:02:01:01	2,905	2,979	0.98	0.97
NATL16	B*51:01:01:05	B*55:02:01:03	3,974	3,468	1.15	1
ATL16	B*51:01:01:05	B*55:02:01:03	1,757	1,598	1.10	0.96
NATL17	B*40:06:01:01	B*56:01:01:03	3,506	3,372	1.04	1
ATL17	B*40:06:01:01	B*56:01:01:03	2,366	2,227	1.06	0.98
NATL18	B*15:01:01:01	-	-	-	-	-
ATL18	B*15:01:01:01	-	-	-	-	-
NATL19	B*52:01:01:02	B*55:02:01:03	3,423	2,754	1.24	1
ATL19	B*52:01:01:02	B*55:02:01:03	3,642	3,086	1.18	0.95
NATL20	B*15:01:01:01	B*35:01:03	3,014	2,609	1.16	1
ATL20	B*15:01:01:01	B*35:01:03	2,630	2,720	0.97	0.84
NATL21	B*48:01:01:01	B*51:01:01:01	4,972	4,898	1.02	1
ATL21	B*48:01:01:01	B*51:01:01:01	5,392	4,987	1.08	0.94
NATL22	B*15:01:01:01	B*35:01:01:51**	4,128	3,769	1.10	1
ATL22	B*15:01:01:01	B*35:01:01:51**	4,863	4,526	1.07	0.98
NATL23	B*35:01:01:02	B*54:01:01:01	2,005	1,674	1.20	1
ATL23	B*35:01:01:02	B*54:01:01:01	5,556	174	31.93	0.04
NATL24	B*07:02:01:01	B*54:01:01:01	3,463	2,722	1.27	1
ATL24	B*07:02:01:01	B*54:01:01:01	5,968	5,119	1.17	0.92
NATL26	B*40:06:01:01	B*54:01:01:01	2,856	2,587	1.10	1
ATL26	B*40:06:01:01	B*54:01:01:01	2,969	3,093	0.96	0.87
NATL27	B*07:02:01:01	B*40:02:01:01	4,130	3,150	1.31	1
ATL27	B*07:02:01:01	B*40:02:01:01	2,888	75	38.51	0.03

Sample ID	HLA-DRB1 Allele name Allele1	HLA-DRB1 Allele name Allele2	HLA-DRB1 Read number Allele1	HLA-DRB1 Read number Allele2	HLA-DRB1 Allele 1 /Allele 2	HLA-DRB1 NR*
NATL01	DRB1*04:06:01	DRB1*08:02:01:01	11,616	19,237	0.60	1
ATL01	DRB1*04:06:01	DRB1*08:02:01:01	11,591	20,359	0.57	0.943
NATL02	DRB1*09:01:02:01	DRB1*15:01:01:03	32,769	27,846	1.18	1
ATL02	DRB1*09:01:02:01	DRB1*15:01:01:03	24,075	17,091	1.41	0.835
NATL03	DRB1*09:01:02:01	DRB1*11:01:01:04	18,024	13,291	1.36	1
ATL03	DRB1*09:01:02:01	DRB1*11:01:01:04	12,929	10,459	1.24	0.91
NATL04	DRB1*09:01:02:01	DRB1*14:05:01:02	20,630	16,404	1.26	1
ATL04	DRB1*09:01:02:01	DRB1*14:05:01:02	18,916	12,531	1.51	0.83
NATL05	DRB1*08:02:01:01	DRB1*15:01:01:03	42,006	26,325	1.60	1
ATL05	DRB1*08:02:01:01	DRB1*15:01:01:03	21,398	17,169	1.25	0.78
NATL06	DRB1*04:05:01:01	DRB1*09:01:02:01	4,299	19,315	0.22	1
ATL06	DRB1*04:05:01:01	DRB1*09:01:02:01	5,304	22,430	0.24	0.94
NATL07	DRB1*14:05:01:02	DRB1*15:01:01:03	22,144	17,221	1.29	1
ATL07	DRB1*14:05:01:02	DRB1*15:01:01:03	9,770	8,341	1.17	0.91
NATL08	DRB1*09:01:02:01	DRB1*15:01:01:03	29,835	22,310	1.34	1
ATL08	DRB1*09:01:02:01	DRB1*15:01:01:03	35,840	26,841	1.34	1.00
NATL09	DRB1*08:03:02:02	DRB1*15:01:01:03	13,605	11,474	1.19	1
ATL09	DRB1*08:03:02:02	DRB1*15:01:01:03	25,910	17,699	1.46	0.81
NATL10	DRB1*09:01:02:01	DRB1*15:01:01:03	48,503	33,367	1.45	1
ATL10	DRB1*09:01:02:01	DRB1*15:01:01:03	30,288	17,031	1.78	0.82
NATL11	DRB1*12:01:01:04	DRB1*13:01:01:01	42,371	43,670	0.97	1
ATL11	DRB1*12:01:01:04	DRB1*13:01:01:01	67,527	83,745	0.81	0.83
NATL12	DRB1*04:05:01:01	DRB1*08:03:02:02	11,426	36,860	0.31	1
ATL12	DRB1*04:05:01:01	DRB1*08:03:02:02	12,327	40,632	0.30	0.98
NATL13	DRB1*04:10:03	DRB1*14:54:01:08**	6,780	21,158	0.32	1
ATL13	DRB1*04:10:03	DRB1*14:54:01:08**	5,128	16,240	0.32	0.99
NATL14	DRB1*08:02:01:01	DRB1*15:01:01:03	14,617	10,683	1.37	1
ATL14	DRB1*08:02:01:01	DRB1*15:01:01:03	16,460	13,013	1.26	0.92
NATL16	DRB1*04:10:01	DRB1*08:02:01:01	11,130	32,317	0.34	1
ATL16	DRB1*04:10:01	DRB1*08:02:01:01	5,432	15,815	0.34	1.00
NATL17	DRB1*04:05:01:01	-	-	-	-	-
ATL17	DRB1*04:05:01:01	-	-	-	-	-
NATL18	DRB1*04:06:01	DRB1*15:01:01:03	6,159	8,379	0.74	1
ATL18	DRB1*04:06:01	DRB1*15:01:01:03	5,273	7,166	0.74	0.999
NATL19	DRB1*15:01:01:03	DRB1*15:02:01:03	3,548	3,339	1.06	1
ATL19	DRB1*15:01:01:03	DRB1*15:02:01:03	3,145	3,147	1.00	0.940
NATL20	DRB1*04:06:01	DRB1*12:01:01:04	9,755	17,084	0.57	1
ATL20	DRB1*04:06:01	DRB1*12:01:01:04	24,685	453	54.49	0.010
NATL21	DRB1*14:54:01:06**	DRB1*15:01:01:03	10,353	7,609	1.36	1
ATL21	DRB1*14:54:01:06**	DRB1*15:01:01:03	7,649	5,625	1.36	0.999
NATL22	DRB1*15:01:01:03	-	-	-	-	-
ATL22	DRB1*15:01:01:03	-	-	-	-	-
NATL23	DRB1*04:05:01:01	DRB1*09:01:02:02	8,270	29,695	0.28	1
ATL23	DRB1*04:05:01:01	DRB1*09:01:02:02	313	41,224	0.01	0.03
NATL24	DRB1*01:01:01:01	DRB1*04:05:01:01	30,371	9,867	3.08	1
ATL24	DRB1*01:01:01:01	DRB1*04:05:01:01	38,765	10,534	3.68	0.84
NATL26	DRB1*04:05:01:01	DRB1*09:01:02:01	8,231	27,546	0.30	1
ATL26	DRB1*04:05:01:01	DRB1*09:01:02:01	5,059	15,969	0.32	0.94
NATL27	DRB1*01:01:01:01	DRB1*14:05:01:02	14,895	14,426	1.03	1
ATL27	DRB1*01:01:01:01	DRB1*14:05:01:02	8,481	6,994	1.21	0.85

Sample ID	HLA-DQA1 Allele name Allele1	HLA-DQA1 Allele name Allele2	HLA-DQA1 Read number Allele1	HLA-DQA1 Read number Allele2	HLA-DQA1 Allele 1 /Allele 2	HLA-DQA1 NR*
NATL01	DQA1*03:01:01:01	-	-	-	-	-
ATL01	DQA1*03:01:01:01	-	-	-	-	-
NATL02	DQA1*01:02:01:01	DQA1*03:02:01:01	5,197	6,028	0.86	1
ATL02	DQA1*01:02:01:01	DQA1*03:02:01:01	4,691	4,559	1.03	0.84
NATL03	DQA1*03:02:01:01	DQA1*05:05:01:09	4,497	4,922	0.91	1
ATL03	DQA1*03:02:01:01	DQA1*05:05:01:09	2,650	2,765	0.96	0.95
NATL04	DQA1*01:04:01:01	DQA1*03:02:01:01	5,652	5,690	0.99	1
ATL04	DQA1*01:04:01:01	DQA1*03:02:01:01	6,585	6,911	0.95	0.96
NATL05	DQA1*01:02:01:01	DQA1*03:01:01:01	6,750	6,462	1.04	1
ATL05	DQA1*01:02:01:01	DQA1*03:01:01:01	4,495	4,487	1.00	0.96

Sample ID	HLA-DQB1 Allele name Allele1	HLA-DQB1 Allele name Allele2	HLA-DQB1 Read number Allele1	HLA-DQB1 Read number Allele2	HLA-DQB1 Allele 1 /Allele 2	HLA-DQB1 NR*
NATL01	DQB1*03:02:01:01	-	-	-	-	-
ATL01	DQB1*03:02:01:01	-	-	-	-	-
NATL02	DQB1*03:01:01:01	DQB1*06:02:01:01	28,353	18,267	1.55	1
ATL02	DQB1*03:01:01:01	DQB1*06:02:01:01	24,862	18,072	1.38	0.89
NATL03	DQB1*03:01:01:03	DQB1*03:03:02:02/03	15,462	16,109	0.96	1
ATL03	DQB1*03:01:01:03	DQB1*03:03:02:02/03	13,093	15,058	0.87	0.91
NATL04	DQB1*03:03:02:02/03	DQB1*05:03:01:02	22,680	27,177	0.83	1
ATL04	DQB1*03:03:02:02/03	DQB1*05:03:01:02	21,865	23,078	0.95	0.88
NATL05	DQB1*03:02:01:01	DQB1*06:02:01:01	33,075	17,290	1.91	1
ATL05	DQB1*03:02:01:01	DQB1*06:02:01:01	24,428	14,186	1.72	0.90

Sample ID	HLA-DPA1 Allele name Allele1	HLA-DPA1 Allele name Allele2	HLA-DPA1 Read number Allele1	HLA-DPA1 Read number Allele2	HLA-DPA1 Allele 1 /Allele 2	HLA-DPA1 NR*
NATL01	DPA1*01:03:01:01	DPA1*02:02:02:02	1,483	1,408	1.05	1
ATL01	DPA1*01:03:01:01	DPA1*02:02:02:02	2,760	2,732	1.01	0.96
NATL02	DPA1*02:01:01:01	DPA1*02:02:02:01	1,947	1,913	1.02	1
ATL02	DPA1*02:01:01:01	DPA1*02:02:02:01	1,595	1,912	0.83	0.82
NATL03	DPA1*01:03:01:01	DPA1*02:02:02:01	2,417	3,293	0.73	1
ATL03	DPA1*01:03:01:01	DPA1*02:02:02:01	2,433	2,354	1.03	0.71
NATL04	DPA1*01:03:01:01	DPA1*01:03:01:08	-	-	-	-
ATL04	DPA1*01:03:01:01	DPA1*01:03:01:08	-	-	-	-
NATL05	DPA1*01:03:01:01	DPA1*02:02:02:01	3,307	2,911	1.14	1
ATL05	DPA1*01:03:01:01	DPA1*02:02:02:01	3,131	3,510	0.89	0.79

Sample ID	HLA-DPA1 Allele name Allele1	HLA-DPA1 Allele name Allele2	HLA-DPA1 Read number Allele1	HLA-DPA1 Read number Allele2	HLA-DPA1 Allele 1 /Allele 2	HLA-DPA1 NR*
NATL01	DPB1*02:01:02:01	DPB1*05:01:01:01	10,215	10,441	0.98	1
ATL01	DPB1*02:01:02:01	DPB1*05:01:01:01	12,737	12,876	0.98	0.99
NATL02	DPB1*05:01:01:01	DPB1*13:01:01:06	8,831	9,312	0.98	1
ATL02	DPB1*05:01:01:01	DPB1*13:01:01:06	6,129	6,746	0.98	0.96
NATL03	DPB1*02:01:02:48**	DPB1*05:01:01:05	10,477	11,279	0.98	1
ATL03	DPB1*02:01:02:48**	DPB1*05:01:01:05	7,414	8,609	0.98	0.93
NATL04	DPB1*02:01:02:11	DPB1*02:01:02:32	-	-	0.98	-
ATL04	DPB1*02:01:02:11	DPB1*02:01:02:32	-	-	0.98	-
NATL05	DPB1*02:01:02:01	DPB1*05:01:01:01	17,527	19,448	0.98	1
ATL05	DPB1*02:01:02:01	DPB1*05:01:01:01	13,171	12,501	0.98	0.86

NATL06	DQA1*03:02:01:01	DQA1*03:03:01:03	963	1,072	0.90	1	DQB1*03:03:02:02/03	DQB1*04:01:01:02	11,535	9,491	1.22	1	DPA1*02:02:02:01	-	-	-	-	-	DPB1*05:01:01:01	DPB1*05:01:01:08	-	-	0.98	-
ATL06	DQA1*03:02:01:01	DQA1*03:03:01:03	1,096	986	1.11	0.81	DQB1*03:03:02:02/03	DQB1*04:01:01:02	13,847	12,198	1.14	0.93	DPA1*02:02:02:01	-	-	-	-	-	DPB1*05:01:01:01	DPB1*05:01:01:08	-	-	0.98	-
NATL07	DQA1*01:02:01:01	DQA1*01:04:01:01	2,920	2,865	1.02	1	DQB1*05:03:01:02	DQB1*06:02:01:01	35,336	15,192	2.33	1	DPA1*02:02:02:01	-	-	-	-	-	DPB1*05:01:01:01	DPB1*05:01:01:18**	-	-	0.98	-
ATL07	DQA1*01:02:01:01	DQA1*01:04:01:01	1,585	1,521	1.04	0.98	DQB1*05:03:01:02	DQB1*06:02:01:01	27,619	12,420	2.22	0.96	DPA1*02:02:02:01	-	-	-	-	-	DPB1*05:01:01:01	DPB1*05:01:01:18**	-	-	0.98	-
NATL08	DQA1*01:02:01:01	DQA1*03:02:01:01	5,415	5,389	1.00	1	DQB1*03:03:02:02/03	DQB1*06:02:01:01	28,113	17,596	1.60	1	DPA1*02:02:02:01	DPA1*02:02:02:11**	-	-	-	-	DPB1*02:01:02:29	DPB1*05:01:01:01	13,776	17,579	0.98	1
ATL08	DQA1*01:02:01:01	DQA1*03:02:01:01	5,642	6,235	0.90	0.90	DQB1*03:03:02:02/03	DQB1*06:02:01:01	43,879	24,217	1.81	0.88	DPA1*02:02:02:01	DPA1*02:02:02:11**	-	-	-	-	DPB1*02:01:02:29	DPB1*05:01:01:01	21,309	20,465	0.98	0.75
NATL09	DQA1*01:02:01:01	DQA1*01:03:01:01	2,039	1,861	1.10	1	DQB1*06:01:01:01	DQB1*06:02:01:01	60,793	30,091	2.02	1	DPA1*02:02:02:01	-	-	-	-	-	DPB1*05:01:01:01	DPB1*05:01:01:05	-	-	0.98	-
ATL09	DQA1*01:02:01:01	DQA1*01:03:01:01	6,015	6,172	0.97	0.89	DQB1*06:01:01:01	DQB1*06:02:01:01	63,100	30,288	2.08	0.97	DPA1*02:02:02:01	-	-	-	-	-	DPB1*05:01:01:01	DPB1*05:01:01:05	-	-	0.98	-
NATL10	DQA1*01:02:01:01	DQA1*03:02:01:01	7,039	5,796	1.21	1	DQB1*03:03:02:02/03	DQB1*06:02:01:01	38,904	18,653	2.09	1	DPA1*02:02:02:01	-	-	-	-	-	DPB1*02:01:02:29	DPB1*05:01:01:01	24,424	9,024	0.98	1
ATL10	DQA1*01:02:01:01	DQA1*03:02:01:01	5,614	6,273	0.89	0.74	DQB1*03:03:02:02/03	DQB1*06:02:01:01	38,647	23,447	1.65	0.79	DPA1*02:02:02:01	-	-	-	-	-	DPB1*02:01:02:29	DPB1*05:01:01:01	28,677	9,038	0.98	0.85
NATL11	DQA1*01:03:01:12**	DQA1*05:06:01:03**	5,352	4,625	1.16	1	DQB1*03:01:01:01	DQB1*06:03:01:01	87,051	49,989	1.74	1	DPA1*01:03:01:05	DPA1*02:02:02:01	15,219	14,005	1.09	1	DPB1*04:02:01:02	DPB1*05:01:01:01	58,550	53,880	0.98	1
ATL11	DQA1*01:03:01:12**	DQA1*05:06:01:03**	7,651	6,044	1.27	0.91	DQB1*03:01:01:01	DQB1*06:03:01:01	59,071	123,704	0.48	0.27	DPA1*01:03:01:05	DPA1*02:02:02:01	16,443	22,328	0.74	0.68	DPB1*04:02:01:02	DPB1*05:01:01:01	67,711	89,385	0.98	0.70
NATL12	DQA1*01:03:01:07	DQA1*03:03:01:03	5,255	5,237	1.00	1	DQB1*04:01:01:02	DQB1*06:01:01:01	27,762	31,713	0.88	1	DPA1*01:03:01:01	DPA1*01:03:01:05	-	-	-	-	DPB1*02:01:02:01	DPB1*04:02:01:02	1,258	1,499	0.98	1
ATL12	DQA1*01:03:01:07	DQA1*03:03:01:03	5,788	6,075	0.95	0.95	DQB1*04:01:01:02	DQB1*06:01:01:01	33,522	37,580	0.89	0.98	DPA1*01:03:01:01	DPA1*01:03:01:05	-	-	-	-	DPB1*02:01:02:01	DPB1*04:02:01:02	2,460	2,525	0.98	0.86
NATL13	DQA1*01:04:01:01	DQA1*03:03:01:02	4,481	5,066	0.88	1	DQB1*04:02:01:05	DQB1*05:03:01:04**	22,272	26,203	0.85	1	DPA1*02:02:02:01	DPA1*02:02:02:02	-	-	-	-	DPB1*03:01:01:08	DPB1*05:01:01:01	11,294	10,989	0.98	1
ATL13	DQA1*01:04:01:01	DQA1*03:03:01:02	4,021	4,315	0.93	0.95	DQB1*04:02:01:05	DQB1*05:03:01:04**	22,464	23,639	0.95	0.89	DPA1*02:02:02:01	DPA1*02:02:02:02	-	-	-	-	DPB1*03:01:01:08	DPB1*05:01:01:01	6,688	5,700	0.98	0.88
NATL14	DQA1*01:02:01:01	DQA1*04:01:01:01	5,404	5,681	0.95	1	DQB1*04:02:01:04	DQB1*06:02:01:01	27,375	12,543	2.18	1	DPA1*01:03:01:08	DPA1*02:02:02:01	2,903	3,053	0.95	1	DPB1*02:01:02:01	DPB1*02:01:02:32	-	-	0.98	-
ATL14	DQA1*01:02:01:01	DQA1*04:01:01:01	4,011	3,925	1.02	0.93	DQB1*04:02:01:04	DQB1*06:02:01:01	23,901	12,884	1.86	0.85	DPA1*01:03:01:08	DPA1*02:02:02:01	2,619	2,714	0.96	0.99	DPB1*02:01:02:01	DPB1*02:01:02:32	-	-	0.98	-
NATL16	DQA1*03:01:01:01	DQA1*03:03:01:01	1,250	1,261	0.99	1	DQB1*03:02:01:01	DQB1*04:02:01:05	13,317	10,858	1.23	1	DPA1*01:03:01:01	DPA1*02:02:02:01	2,607	3,329	0.78	1	DPB1*03:01:01:09	DPB1*47:01:01:01	5,746	5,979	0.98	1
ATL16	DQA1*03:01:01:01	DQA1*03:03:01:01	597	529	1.13	0.88	DQB1*03:02:01:01	DQB1*04:02:01:05	6,761	6,931	0.98	0.80	DPA1*01:03:01:01	DPA1*02:02:02:01	1,932	2,193	0.88	0.89	DPB1*03:01:01:09	DPB1*47:01:01:01	8,621	9,128	0.98	0.98
NATL17	DQA1*03:03:01:03	-	-	-	-	-	DQB1*04:01:01:02	-	-	-	-	-	DPA1*01:03:01:08	DPA1*02:02:02:01	2,342	2,470	0.95	1	DPB1*02:01:02:32	DPB1*05:01:01:01	15,082	11,753	0.98	1
ATL17	DQA1*03:03:01:03	-	-	-	-	-	DQB1*04:01:01:02	-	-	-	-	-	DPA1*01:03:01:08	DPA1*02:02:02:01	2,059	1,866	1.10	0.86	DPB1*02:01:02:32	DPB1*05:01:01:01	7,340	6,866	0.98	0.83
NATL18	DQA1*01:02:01:01	DQA1*03:01:01:01	5,942	5,280	1.13	1	DQB1*03:02:01:01	DQB1*06:02:01:01	31,843	17,029	1.87	1	DPA1*01:03:01:01	-	-	-	-	-	DPB1*02:01:02:01	-	-	-	0.98	-
ATL18	DQA1*01:02:01:01	DQA1*03:01:01:01	2,384	2,381	1.00	0.89	DQB1*03:02:01:01	DQB1*06:02:01:01	10,829	7,909	1.37	0.73	DPA1*01:03:01:01	-	-	-	-	-	DPB1*02:01:02:01	-	-	-	0.98	-
NATL19	DQA1*01:02:01:01	DQA1*01:03:01:01	3,923	3,456	1.14	1	DQB1*06:01:01:01	DQB1*06:02:01:01	25,083	16,283	1.54	1	DPA1*01:03:01:01	DPA1*02:01:01:02	1,261	1,223	1.03	1	DPB1*02:01:02:01	DPB1*09:01:01	18,544	19,702	0.98	1
ATL19	DQA1*01:02:01:01	DQA1*01:03:01:01	5,381	4,192	1.28	0.88	DQB1*06:01:01:01	DQB1*06:02:01:01	36,142	22,096	1.64	0.94	DPA1*01:03:01:01	DPA1*02:01:01:02	3,527	4,248	0.83	0.81	DPB1*02:01:02:01	DPB1*09:01:01	15,566	15,619	0.98	0.94
NATL20	DQA1*03:01:01:01	DQA1*05:06:01:01	4,816	4,275	1.13	1	DQB1*03:01:01:01	DQB1*03:02:01:01	17,000	19,286	0.88	1	DPA1*01:03:01:01	DPA1*02:01:01:02	1,777	1,744	1.02	1	DPB1*02:01:02:01	DPB1*14:01:01:01	10,133	8,729	0.98	1
ATL20	DQA1*03:01:01:01	DQA1*05:06:01:01	7,963	126	63.20	0.02	DQB1*03:01:01:01	DQB1*03:02:01:01	564	42,978	0.01	0.01	DPA1*01:03:01:01	DPA1*02:01:01:02	7,026	111	63.30	0.02	DPB1*02:01:02:01	DPB1*14:01:01:01	23,828	45	0.98	0.00
NATL21	DQA1*01:02:01:01	DQA1*01:04:01:01	2,289	2,202	1.04	1	DQB1*05:02:01:03	DQB1*06:02:01:01	26,697	12,821	2.08	1	DPA1*01:03:01:01	DPA1*01:03:01:05	-	-	-	-	DPB1*02:01:02:01	DPB1*04:02:01:02	1,770	1,682	0.98	1
ATL21	DQA1*01:02:01:01	DQA1*01:04:01:01	2,539	2,412	1.05	0.99	DQB1*05:02:01:03	DQB1*06:02:01:01	34,641	16,671	2.08	1.00	DPA1*01:03:01:01	DPA1*01:03:01:05	-	-	-	-	DPB1*02:01:02:01	DPB1*04:02:01:02	1,803	1,813	0.98	0.95
NATL22	DQA1*01:02:01:01	-	-	-	-	-	DQB1*06:02:01:01	-	-	-	-	-	DPA1*02:01:01:01	DPA1*02:02:02:01	1,462	1,199	1.22	1	DPB1*02:01:02:29	DPB1*13:01:01:06	13,465	13,253	0.98	1
ATL22	DQA1*01:02:01:01	-	-	-	-	-	DQB1*06:02:01:01	-	-	-	-	-	DPA1*02:01:01:01	DPA1*02:02:02:01	1,776	1,990	0.89	0.73	DPB1*02:01:02:29	DPB1*13:01:01:06	14,851	14,444	0.98	0.99
NATL23	DQA1*03:02:01:01	DQA1*03:03:01:03	1,216	954	1.27	1	DQB1*03:03:02:02/03	DQB1*04:01:01:02	13,112	12,088	1.08	1	DPA1*02:01:01:13**	DPA1*02:02:02:01	985	1,055	0.93	1	DPB1*05:01:01:01	-	-	-	0.98	-
ATL23	DQA1*03:02:01:01	DQA1*03:03:01:03	2,640	89	29.66	0.04	DQB1*03:03:02:02/03	DQB1*04:01:01:02	43,228	1,498	28.86	0.04	DPA1*02:01:01:13**	DPA1*02:02:02:01	3,593	184	19.53	0.05	DPB1*05:01:01:01	-	-	-	0.98	-
NATL24	DQA1*01:01:01:04	DQA1*03:03:01:03	3,763	3,426	1.10	1	DQB1*04:01:01:02	DQB1*05:01:01:03	17,093	18,679	0.92	1	DPA1*01:03:01:05	-	-	-	-	-	DPB1*04:02:01:02	-	-	-	0.98	-
ATL24	DQA1*01:01:01:04	DQA1*03:03:01:03	5,225	5,571	0.94	0.85	DQB1*04:01:01:02	DQB1*05:01:01:03	31,876	36,472	0.87	0.96	DPA1*01:03:01:05	-	-	-	-	-	DPB1*04:02:01:02	-	-	-	0.98	-
NATL26	DQA1*03:02:01:01	DQA1*03:03:01:03	1,180	1,346	0.88	1	DQB1*03:03:02:02/03	DQB1*04:01:01:02	12,875	11,079	1.16	1	DPA1*02:02:02:01	-	-	-	-	-	DPB1*05:01:01:01	DPB1*05:01:01:05	-	-	0.98	-
ATL26	DQA1*03:02:01:01	DQA1*03:03:01:03	1,113	1,010	1.10	0.80	DQB1*03:03:02:02/03	DQB1*04:01:01:02	11,481	9,997	1.15	0.99	DPA1*02:02:02:01	-	-	-	-	-	DPB1*05:01:01:01	DPB1*05:01:01:05	-	-	0.98	-
NATL27	DQA1*01:01:01:04	DQA1*01:04:01:01	640	797	0.80	1	DQB1*05:01:01:03	DQB1*05:03:01:01	8,614	11,122	0.77	1	DPA1*01:03:01:05	DPA1*02:02:02:02	3,920	3,563	1.10	1	DPB1*04:02:01:02	DPB1*05:01:01:01	13,923	11,458	0.98	1
ATL27	DQA1*01:01:01:04	DQA1*01:04:01:01	430	446	0.96	0.83	DQB1*05:01:01:03	DQB1*05:03:01:01	5,628	5,508	1.02	0.76	DPA1*01:03:01:05	DPA1*02:02:02:02	1,416	1,318	1.07	0.98	DPB1*04:02:01:02	DPB1*05:01:01:01	4,848	4,681	0.98	0.85

*NR indicates relative depth ratio of ATL cells (ATL) to non-ATL cells (NATL). **HLA alleles shown by red letter indicate novel alleles identified in both of ATL and non-ATL cells. Gray background indicates homozygous HLA genotype.

Supplementary Table 7. Detection of lower values than the confidence interval using relative depth ratios of ATL to non-ATL cells in HLA genes

Sample ID	HLA-A	HLA-B	HLA-C	HLA-DRB1	HLA-DQA1	HLA-DQB1	HLA-DPA1	HLA-DPB1
ATL 01	**0.17**	**0.15**	**0.15**	0.94	-	-	0.96	0.99
ATL 02	0.92	0.94	0.87	0.84	0.84	0.89	0.82	0.96
ATL 03	0.91	0.88	0.90	0.91	0.95	0.91	0.71	0.93
ATL 04	0.98	0.86	0.81	0.83	0.96	0.88	-	-
ATL 05	0.98	0.96	0.80	0.78	0.96	0.90	0.79	0.86
ATL 06	0.97	1.00	0.91	0.94	0.81	0.93	-	-
ATL 07	0.94	0.99	1.00	0.91	0.98	0.96	-	-
ATL 08	**0.01**	**0.02**	**0.01**	1.00	0.90	0.88	-	0.75
ATL 09	-	0.93	0.83	0.81	0.89	0.97	-	-
ATL 10	0.96	0.99	0.98	0.82	0.74	0.79	-	0.85
ATL 11	**0.01**	0.71	0.66	0.83	0.91	**0.27**	0.68	0.70
ATL 12	1.00	0.98	0.94	0.98	0.95	0.98	-	0.86
ATL 13	**0.01**	**0.01**	**0.02**	0.99	0.95	0.89	-	0.88
ATL 14	0.95	0.97	-	0.92	0.93	0.85	0.99	-
ATL 16	-	0.96	0.94	1.00	0.88	0.80	0.89	0.98
ATL 17	0.90	0.98	0.94	-	-	-	0.86	0.83
ATL 18	0.99	-	0.84	1.00	0.89	0.73	-	-
ATL 19	**0.03**	0.95	0.95	0.94	0.88	0.94	0.81	0.94
ATL 20	0.85	0.84	0.86	**0.01**	**0.02**	**0.01**	**0.02**	**0.00**
ATL 21	0.96	0.94	0.94	1.00	0.99	1.00	-	0.95
ATL 22	0.90	0.98	0.92	-	-	-	0.73	0.99
ATL 23	**0.03**	**0.04**	**0.03**	**0.03**	**0.04**	**0.04**	**0.05**	-
ATL 24	0.92	0.92	0.97	0.84	0.85	0.96	-	-
ATL 26	0.76	0.87	0.88	0.94	0.80	0.99	-	-
ATL 27	-	**0.03**	**0.03**	0.85	0.83	0.76	0.98	0.85
Sample number	22	24	24	23	22	22	13	16
Average	0.69	0.74	0.72	0.83	0.82	0.79	0.71	0.83
Standard deviation	0.41	0.37	0.36	0.27	0.26	0.29	0.32	0.24
t(Confidence coefficient: 99%)	0.25	0.21	0.21	0.16	0.16	0.17	0.27	0.17
Confidence interval	0.44-0.93	0.53-0.96	0.51-0.92	0.67-0.99	0.66-.097	0.61-0.96	0.44-0.98	0.66-1.01

HLA alleles shown by red letter indicate lower values than the confidence interval. Gray background indicates homozygous HLA genotype.

Supplementary Table 8. Primer information used for comfirmation of HLA-LOH

HLA locus	Primer name	Primer sequence (5' to 3')	Amplified region	Analyzed exon	Reference
HLA-A	AF	GAAACSGCCTCTGYGGGGAGAAGCAA	Exon 2-3	Exons 2, 3	J Immunol Methods. 382: 40-47, 2012
	AR	TGTTGGTCCCAATTGTCTCCCCTC			
	AS1F	AGCCGCGCCKGGASGAGGGTC			
	AS4R	TGTGGGAGGCCAGCCCGGGAGA			
HLA-B	BF_mod	GGGAGGAGMRAGGGGACCSCAG	Exon 2-3	Exons 2, 3	J Immunol Methods. 382: 40-47, 2012
	BR	GGAGGCCATCCCCGGCGACCTAT			
	BnewF	GCAGGCGGGGGCGCAGGACC			
	BS4R	GGAGATGGGGAAGGCTCCCCACT			
HLA-C	CF	ARCGAGGKGCCCKCCCGGCGA	Exon 2-3	Exons 2, 3	J Immunol Methods. 382: 40-47, 2012
	CS1F	GGAGCCGCGCAGGGAGGWGGGTC			
	CS7R	GGCTCCCCACTGCCCYTGGTAC			
HLA-DRB1	DRB1-52.1	CCCACAGCACGTTTCTTGGAGTACYCTA	Exon 2	Exon 2	Tissue Antigens. 63: 412-23, 2004
	DRB1-09	CAGCACGTTTCTTGAAGCAGGATAAGTT			
	DRB1-04	GCACGTTTCTTGGAGCAGGTTAAAC			
	DRB-R	TGCTYACCTCGCCKCTGCAC			
HLA-DQA1	DQA1_e1_F2	GTTTGGGTGTCTTCAGATT	Exon 1-3	Exons 1, 2, 3	This study
	DQA1_e3_R2	AGGCAGGAAGTTCTGAAC			
HLA-DQB1	DQB1_346_F1	GACTGACCGGCCGGTGATTCCC	Exon 2	Exon 2	This study
	DQB1_346_R	CAACTGTAATTGCTGGATGCCCTGCGC			
HLA-DPA1	DPA1-seq11	CACTTGCATATTCAAACTGA	Exon 2	Exon 2	This study
	DPA1-seq1	CACTCTGCTCACCTTTCTCT			
HLA-DPB1	DPB1_F2	GCTCCCTTTAGCGAGTCCTTC	Exon 1-4	Exons 2, 3	This study
	DPB1-R2	TCAGCCATTGAAACCACCTC			

Supplementary Table 9. Confirmation of HLA-LOH by Sanger direct-sequencing

HLA locus	non-ATL			ATL		
	Sample ID	Allele 1	Allele 2	Sample ID	Allele 1	Allele 2
A	NATL01	A*11:01	A*26:01	ATL01	A*11:01	A*26:01
	NATL08	A*02:06	A*31:01	ATL08	A*31:01	/
	NATL11	A*11:01	A*24:02	ATL11	A*11:01	/
	NATL13	A*02:06	A*24:02	ATL13	A*02:06	/
	NATL19	A*24:02	A*31:01	ATL19	A*24:02	/
	NATL23	A*24:02	A*31:01	ATL23	A*31:01	/
B	NATL01	B*15:01	B*40:02	ATL01	B*15:01	/
	NATL08	B*35:01	B*56:01	ATL08	B*56:01	/
	NATL13	B*35:01	B*40:06	ATL13	B*40:06	/
	NATL23	B*35:01	B*54:01	ATL23	B*35:01	/
	NATL27	B*07:02	B*40:02	ATL27	B*07:02	/
C	NATL01	C*03:04	C*04:01	ATL01	C*04:01	/
	NATL08	C*03:03	C*04:01	ATL08	C*04:01	/
	NATL13	C*03:03	C*15:02	ATL13	C*15:02	/
	NATL23	C*01:02	C*03:03	ATL23	C*03:03	/
	NATL27	C*03:03	C*07:02	ATL27	C*07:02	/
DRB1	NATL20	DRB1*04:06	DRB1*12:01	ATL20	DRB1*04:06	/
	NATL23	DRB1*04:05	DRB1*09:01	ATL23	DRB1*09:01	/
DQA1	NATL20	DQA1*03:01	DQA1*05:06	ATL20	DQA1*03:01	/
	NATL23	DQA1*03:02	DQA1*03:03	ATL23	DQA1*03:02	/
DQB1	NATL11	DQB1*03:01	DQB1*06:03	ATL11	DQB1*03:01	DQB1*06:03
	NATL20	DQB1*03:01	DQB1*03:02	ATL20	DQB1*03:02	/
	NATL23	DQB1*03:03	DQB1*04:01	ATL23	DQB1*03:03	/
DPA1	NATL20	DPA1*01:03	DPA1*02:01	ATL20	DPA1*01:03	/
	NATL23	DPA1*02:01	DPA1*02:02	ATL23	DPA1*02:01	/
DPB1	NATL20	DPB1*02:01	DPB1*14:01	ATL20	DPB1*02:01	/

Red letter indicates sample ID and HLA genotype assigned to the heterozygotes. NATL indicates non-ATL cell.

Supplementary Table 10: MFI ratio of HLA class I in patients with HLA-LOH and/or NSVs and in those without HLA-LOH and/or NSVs.

Patients without HLA-LOH/NSVs				Patients with HLA-LOH/NSVs			
Paitent	MFI of HLA class I		MFI ratio	Paitent	MFI of HLA class I		MFI ratio
	Non-ATL cells	ATL cells			Non-ATL cells	ATL cells	
ATL02	5369	9672	1.80	ATL01	7899	5848	0.74
ATL03	5016	5896	1.18	ATL19	7466	4523	0.61
ATL04	10346	11181	1.08	ATL20	11583	743	0.06
ATL05	9368	7286	0.78	ATL23	5784	4385	0.76
ATL06	8841	14394	1.63	ATL27	11042	3218	0.29
ATL07	6002	7249	1.21				
ATL17	6338	7204	1.14				
ATL21	3934	8877	2.26				
ATL26	4052	9127	2.25				

MFI indicates mean fluorescence intensity and MFI ratio indicates MFI ratio indicates relative MFI ratio of ATL cells to non-ATL cells. HLA-LOH indicates NSVs indicates non-silent varitants.
