# Supplementary material for: Full-length HLA sequencing in adult T cell leukemia–lymphoma uncovers multiple gene alterations
Source: Leukemia. 2021 Sep 13;35(10):2998–3001. doi: 10.1038/s41375-021-01403-1 (PMC8478651; doi:10.1038/s41375-021-01403-1)
Supplement: Supplementary file 3 — Supplementary methods [file 41375_2021_1403_MOESM3_ESM.pdf]

## Supplementary Methods

### *Separation of ATL cells and non-ATL cells*

PBMCs were stained with fluorescein isothiocyanate (FITC)-conjugated anti-CADM1 antibody (MBL, Nagoya, Japan), phycoerythrin-cyanin 7 (PE/Cy7)-conjugated anti-CD4 antibody (BioLegend, San Diego, CA), and allophycocyanin (APC)-conjugated anti-CD25 antibody (BioLegend). After being washed, the cells were incubated with anti-FITC MACS beads (Miltenyi Biotec, Bergisch Gladbach, Germany), and then cell separation was conducted using a MACS cell separation kit (Miltenyi Biotec). Flow cytometric analysis was performed using a BD FACSverse (BD Biosciences), and the data were analyzed using FlowJo software (TOMY Digital Biology, Tokyo, Japan). Genomic DNA was extracted from CADM1-positive ATL cells and CADM1-negative non-ATL cells using a QIAamp DNA Mini Kit (QIAGEN, Hilden, Germany).

### *NGS using the Ion Torrent system*

Entire gene regions from the promoter-enhancer region to the 3'UTR of HLA-A, HLA-B, HLA-C, HLA-DRB1, HLA-DQA1, HLA-DQB1, HLA-DPA1, and HLA-DPB1 were independently amplified by a previously developed long-range PCR method11 using genomic DNA obtained from CADM1-positive ATL cells and CADM1-negative non-ATL cells. Preparation of barcoded library DNA samples from the amplicons, fragmentation, DNA library amplification, measurement of DNA size, and quantitation were performed as in a previous report [1]. Each barcoded library was mixed at equimolar concentrations and then diluted according to the manufacturer's recommendations. Emulsion PCR was performed with the Ion 520 & 530 Kit-OT2 and Ion OneTouch 2 instrument (Thermo Fisher Scientific). Next, the beads carrying single-stranded DNA templates were enriched with the Ion OneTouch Enrichment System (Thermo Fisher Scientific) according to the manufacturer's recommendations. Sequencing was performed using the Ion S5 Sequencing Kit and Ion 520/530 Chip Kit (Thermo Fisher Scientific).

### *Raw data processing after NGS run*

Raw data processing after NGS run such as base-calling, trimming and output of quality-filter sequence reads that were binned on the basis of the Ion Xpress Barcodes into separate sequence fastq files, were all performed with Torrent Suite 5.12.1 (Thermo Fisher Scientific) for the genotyping with full processing for shotgun analysis. These sequence data files were further quality trimmed to remove poor sequences at the end of the reads with quality values (QVs) of less than 15. The QVs of the sequence reads were calculated by the fastx_quality_stats included with the FASTX-Toolkit for short-reads data preprocessing (http://hannonlab.cshl.edu/fastx_toolkit/).

### *HLA allele assignment*

The trimmed and barcode-binned fastq files for each sample were used for genotyping and allele assignment up to the field-3 level (an allele resolution in which alleles are defined by synonymous and/or nonsynonymous DNA substitutions in the coding region). The HLA alleles for the eight classical HLA loci were assigned by nucleotide similarity searches in the IPD-IMGT/HLA database (https://www.ebi.ac.uk/ipd/imgt/hla/) using the BLAT program [2], included in our in-house Sequence Alignment Based Assigning Software (SeaBass) [3].

### *Discovery of germline and somatic mutations*

Mapping of the reads from non-ATL cells and the full-length HLA sequences assigned by the BLAT search as references was performed using GS Reference Mapper version 3.0 software (Roche, Basel, Switzerland). The mapping parameter was set to a 100% matched condition between the reads and the references to avoid mis-mapping among the HLA loci and contamination of in vitro-generated PCR crossover products. If the reads were not mapped to a reference, then we used a consensus sequence output from GS Reference Mapper for secondary mapping to generate a reference sequence. During this process, when we detected germline mutations in a consensus sequence, we artificially modified the sequence and used it as a tentative reference for read mapping. After construction of the references from non-ATL cells, we detected novel mutation candidates by mapping reads from ATL cells to the references using the above-indicated mapping parameters. Finally, all of the mutation candidates were identified as germline mutations, somatic mutations, or sequence errors by Sanger direct-sequencing using newly designed sequencing primers and genomic DNA obtained from ATL and non-ATL cells.

### *Detection of LOH generated in ATL cells*

After the reads were mapped, the separate reads for each allele were used to calculate the average depth ratio between alleles, which is an indicator of LOH presence. The average depth ratio was calculated for ATL cells and non-ATL cells as the “read numbers of one allele/read numbers of the other allele”. We then calculated the normalized average depth ratios (NRs) as the relative depth ratio of ATL cells to non-ATL cells (Supplementary Methods). The NR has a maximum of 1.0, with 1.0 indicating that the average depth ratios of ATL cells and non-ATL cells are identical. Mean values of the NRs for each locus were calculated, and LOH was considered to be present if the NR showed less than the lower bound of the 99.9% confidence interval for the mean value. Loss of HLA haplotypes (allelic loss combinations) were inferred using the Japanese HLA haplotype frequency database [4].

### *Sanger direct-sequencing using the ABI3130xl genetic analyzer*

Purified PCR amplicons were sequenced bidirectionally, to validate newly discovered single nucleotide variations (SNVs) and indels with the Big Dye Terminator Kit v.1.1 (Thermo Fisher Scientific) using the

ABI3130xl genetic analyzer (Thermo Fisher Scientific). The generated chromatogram sequence data were analyzed with Sequencher version 5.0.1 DNA sequence assembly software (Gene Code, Ann Arbor, MI).

### *Confirmation of HLA-LOH by Sanger direct-sequencing*

The HLA-A, HLA-B and HLA-C specific primer sets[5] and HLA-DRB1 specific primer sets[6] were used to confirm the presence of HLA-LOH of HLA-A, HLA-B and HLA-C genes obtained from the NGS analysis (Supplementary Table 8). The primers targeted exons 2 to 3 in HLA-A, HLA-B and HLA-C and exon 2 in HLA-DRB1 to amplify the genes. The HLA-DQA1, HLA-DQB1, HLA-DPA1 and HLA-DPB1 specific primer sets were newly designed and used to confirm the presence of HLA-LOH of HLA-DQA1, HLA-DQB1, HLA-DPA1 and HLA-DPB1 genes (Supplementary Table 8). The primers targeted exon 2 in HLA-DQB1 and HLA-DPA1, exons 1 to 4 in HLA-DQA1 and exons 2 to 3 in HLA-DPB1 to amplify the genes. The 20 μL amplification-reaction-volume contained 10 ng of genomic DNA, 1 unit of PrimeSTAR GXL DNA polymerase (TaKaRa Bio.), 4.0 μL of 5 × PrimeSTAR GXL Buffer (5 mM $Mg2^{+}$), 1.6 μL of 2.5 mM of each dNTP and 1 μL (10 pmol/μL) of each primer. The cycling parameters were as follows: an initial denaturation 94˚C/2 min., followed by 30 cycles for 98˚C/10 sec. 60˚C/30 sec. and 68˚C/3 min (HLA-DQA1), for 98˚C/10 sec. and 68˚C/1 min. (HLA-DQB1), for 98˚C/10 sec. 58˚C/15 sec. and 68˚C/1 min. (HLA-DPA1), and for 98˚C/10 sec. and 68˚C/3 min. (HLA-DPB1). The composition of reaction mixtures and PCR condition of HLA-A, HLA-B, HLA-C and HLA-DRB1 were performed according to the previously published methods[5, 6]. The PCR products were purified by the QIAquick PCR Purification Kit (QIAGEN) and determined the nucleotide sequences by Sanger direct-sequencing method.

### *Flow cytometric analysis of HLA class I expression*

PBMCs were stained with FITC-conjugated anti-CADM1 antibody, PE/Cy7-conjugated anti-CD4 antibody, PerCP/Cyanine5.5-conjugated anti-CD3 antibody (BioLegend), and Pacific Blue-conjugated anti-human HLA class I antibody (BioLegend). Flow cytometric analysis was performed using BD FACSverse (BD Biosciences), and data were analyzed using FlowJo software.

### *Statistical analysis*

Disease-related factors were compared between acute type ATL patients without loss of heterozygosity in HLA genes (HLA-LOH) and/or non-silent variants (NSVs) and those without HLA-LOH/St-mts using the Fisher's exact test for categorical variables and the Mann-Whitney U test for continuous variables. For analysis of overall survival (OS), OS was defined as the time from date of diagnosis to date of last follow-up or date of death. Survival curves were estimated using the Kaplan-Meier method and compared by the log-rank test. Mean fluorescence intensity (MFI) ratios of HLA class I were compared between patients with and without HLA-LOH/NSVs using the Mann–Whitney U test.

***Workflow for calculating normalized average depth ratios (NRs) from sequence reads.***

(A) shows the positions of the region that was subjected to long-range PCR (brown arrow) and of the region used for calculating the LOH detection (blue arrow) at the eight HLA loci by the SS-SBT method. In the case of class I loci, all coding sequences and introns between them were used for LOH detection. On the other hand, in the case of class II loci, the coding sequences after exon 2 and the introns between them were used for LOH detection because the nucleotide length of the regions is relatively uniform among the alleles of each HLA locus. Pink, white, and black boxes indicate the promoter/enhancer region, coding sequence, and untranslated region, respectively. The number on the box indicates the exon number. (B) shows read numbers mapped for the applicable HLA alleles of HLA-A, HLA-B, and HLA-C loci from the reads of case 1 (ATL01 and NATL01) as an example (see Supplementary Table 6). (C) and (D) show methods for calculating the relative depth ratio between alleles and the normalized relative depth ratio (NR) between ATL and non-ATL cells, respectively. The tables show an example of the calculation methods that use the read numbers described in Supplementary Method (B). The red letter in (D) indicates the finally generated NR value used for the comparison among ATL cases described in Supplementary Table 7.

**Figure for Supplementary Method (Workflow of normalized average depth ratios (NRs) from the sequence reads)**

(A)

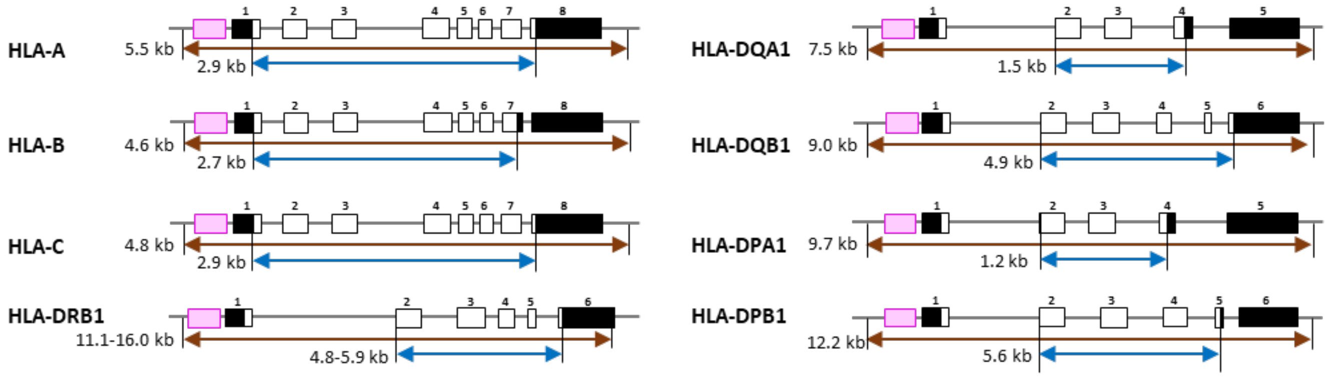


(B)

| HLA locus | | HLA-A | | HLA-C | | HLA-B | |
|---|---|---|---|---|---|---|---|
| Allele 1 / Allele 2 | | Allele1 | Allele2 | Allele1 | Allele2 | Allele1 | Allele2 |
| HLA allele | | A*11:01:01:01 | A*26:01:01:01 | C*03:04:01:02 | C*04:01:01:01 | B*15:01:01:01 | B*40:02:01:01 |
| Sample ID | NATL01 | 5,645 | 6,753 | 5,420 | 5,360 | 5,699 | 5,258 |
| | ATL01 | 1,388 | 9,886 | 1,296 | 8,790 | 7,493 | 1,012 |

(C) The relative depth ratio was calculated by below formula.

**Relative depth ratio = read number of allele 1 / read number of allele 2**

| HLA locus | | HLA-A | HLA-C | HLA-B |
|---|---|---|---|---|
| Sample ID | NATL01 | 5,645 / 6,753 = **0.84** | 5,420 / 5,360 = **1.01** | 5,699 / 5,258 = **1.08** |
| | ATL01 | 1,388 / 9,886 = **0.14** | 1,296 / 8,790 = **0.15** | 7,493 / 1,012 = **7.40** |

(D) The normalized relative depth ratio (NR) was calculated by below formula.

**NR = 1 / depth ratio of NATL x depth ratio of ATL**

In case of showing more than 1.0 NR, we correct the number to less than 1.0 NR.

**NR (<1.0) = 1 / NR (>1.0)**

| HLA locus | HLA-A | HLA-C | HLA-B |
|---|---|---|---|
| NR of ATL01 | **0.17** | **0.15** | **0.15** |

## References for Supplementary Methods


1. Suzuki S, Ranade S, Osaki K, Ito S, Shigenari A, Ohnuki Y, et al. Reference Grade Characterization of Polymorphisms in Full-Length HLA Class I and II Genes With Short-Read Sequencing on the ION PGM System and Long-Reads Generated by Single Molecule, Real-Time Sequencing on the PacBio Platform. *Front Immunol.* 2018;9:2294.
2. Kent WJ. BLAT--the BLAST-like alignment tool. *Genome Res.* 2002;12:656-664.
3. Shiina T, Suzuki S, Kulski JK, Inoko H. Super High Resolution for Single Molecule-Sequence-Based Typing of Classical HLA Loci Using Ion Torrent PGM. *Methods Mol Biol.* 2018;1802:115-133.
4. Ikeda N, Kojima H, Nishikawa M, Hayashi K, Futagami T, Tsujino T, et al. Determination of HLA-A, -C, -B, -DRB1 allele and haplotype frequency in Japanese population based on family study. *Tissue Antigens.* 2015;85:252-259.
5. Cotton LA, Abdur Rahman M, Ng C, Le AQ, Milloy MJ, Mo T, et al. HLA class I sequence-based typing using DNA recovered from frozen plasma. *J Immunol Methods.* 2012;382:40-47.
6. Sayer DC, Whidborne R, De Santis D, Rozemuller EH, Christiansen FT, Tilanus MG. A multicenter international evaluation of single-tube amplification protocols for sequencing-based typing of HLA-DRB1 and HLA-DRB3,4,5. *Tissue Antigens.* 2004;63:412-423.
